# Heritable genome-wide variation of gene expression and promoter methylation between wild and domesticated chickens

**DOI:** 10.1186/1471-2164-13-59

**Published:** 2012-02-04

**Authors:** Daniel Nätt, Carl-Johan Rubin, Dominic Wright, Martin Johnsson, Johan Beltéky, Leif Andersson, Per Jensen

**Affiliations:** 1 IFM Biology, Division of Zoology, Avian Behavioural Genomics and Physiology Group, Linköping University, Sweden; 2 Department of Medical Biochemistry and Microbiology, Uppsala University, Sweden

**Keywords:** Domestication, gene expression, tiling array, behaviour, methylation

## Abstract

**Background:**

Variations in gene expression, mediated by epigenetic mechanisms, may cause broad phenotypic effects in animals. However, it has been debated to what extent expression variation and epigenetic modifications, such as patterns of DNA methylation, are transferred across generations, and therefore it is uncertain what role epigenetic variation may play in adaptation.

**Results:**

In Red Junglefowl, ancestor of domestic chickens, gene expression and methylation profiles in thalamus/hypothalamus differed substantially from that of a domesticated egg laying breed. Expression as well as methylation differences were largely maintained in the offspring, demonstrating reliable inheritance of epigenetic variation. Some of the inherited methylation differences were tissue-specific, and the differential methylation at specific loci were little changed after eight generations of intercrossing between Red Junglefowl and domesticated laying hens. There was an over-representation of differentially expressed and methylated genes in selective sweep regions associated with chicken domestication.

**Conclusions:**

Our results show that epigenetic variation is inherited in chickens, and we suggest that selection of favourable epigenomes, either by selection of genotypes affecting epigenetic states, or by selection of methylation states which are inherited independently of sequence differences, may have been an important aspect of chicken domestication.

## Background

Chickens were domesticated from the Red Junglefowl (RJF) about 8000 years ago [1,2], and the changes in morphology, physiology and behaviour as a response to this have been immense. For example, most domesticated chickens grow to at least twice the size of RJF, become sexually mature at a lower age, lay manifold more and larger eggs, show a wide variation in plumage colour and structure, and have a different behaviour in a number of contexts, such as reduced fearfulness [3-6]. In general, domestic animals are assumed to have adapted to a life among humans by evolving higher flexibility in diet, better ability to breed in captivity, less stress susceptibility, and a more socially tolerant disposition [5,6]. It has been suggested that epigenetic mechanisms might be involved in cases like this [7] where wide-encompassing phenotypic changes occur in a short evolutionary time.

However, there is limited knowledge of the extent to which expression and epigenetic profiles are inherited in animals. Reliable inheritance is necessary in order for epigenetic variation to be a major component of any evolutionary process. We have earlier shown that stress-induced modifications in both behaviour and brain gene expression profiles in domestic chickens are to some extent transferred to the offspring [8,9], and other studies have shown similar transgenerational transmission in other species, including humans [10-12]. This indicates that some epigenetic variation may indeed be inherited, but the details and significance of this, as well as its putative evolutionary significance, remain to be elucidated.

One of the possible epigenetic mechanisms, which could be related to variation in gene expression, is methylation of cytosine, preferentially in so called CpG-islands of promoter regions [13,14]. Therefore, we targeted methylation and gene expression simultaneously to investigate whether any of those, or both, would differ between two populations of chickens, recently separated by domestication. We hypothesised that both methylation and gene expression would differ between the populations and show transgenerational stability, opening the possibility for both to be involved in domestication-related phenotypic changes.

By using expression and methylation arrays on hypothalamus samples, we show that profiles of gene expression as well as promoter methylation differ between domesticated White Leghorn layer chickens and their ancestors, the Red Junglefowl. There were also similar differences, although less pronounced, between phenotypically different families within breeds. The differences were largely maintained in the offspring, demonstrating a reliable inheritance of epigenetic states, and for some of the genes the differential methylation was maintained after eight generations of intercross. Our results therefore suggest that selection of favourable epigenetic variants may have been an important aspect of chicken domestication.

## Results and discussion

### Brain gene expression differences within and between populations

In this experiment, we studied variations in gene expression and methylation in brains of RJF and domesticated White Leghorns (WL), and their offspring. We focussed on thalamus and hypothalamus, brain regions involved in fear and stress responses, both of which have changed significantly during domestication [3,6]. Within each population, we selected parental animals with divergent phenotypes in order to maximise the within population genetic variation. Specifically, we used two pairs of each population, with pairs within population differing in their behaviour in a series of previously validated tests of stress reactions in chickens [6,15]. From these, totally 73 offspring were hatched and reared until three weeks of age, when they were tested in a fear test, similar to that used in the parents.

In both breeds, body weights differed between families in both generations, and behavioural scores, as measured in the fear tests, differed between families in both generations of WL, but not RJF (Additional file 1). Hence, morphological, and to some extent behavioural, phenotypes showed a significant and transgenerationally stable variation in the animals used for the present study. It should be noted that phenotyping was done at different ages in the two generations, which may have been the reason for the lack of transgenerational correlation in fear behaviour in RJF. All eight parents were sacrificed at an age of 373 days, and 48 offspring (12 from each pair) at 21 days, and from each brain, the thalamus-hypothalamus region was removed for extraction of both DNA and mRNA. For the offspring, eight pools of both were prepared, each consisting of six same-sex samples within families. Hence, there were in total eight parental single-animal samples, and eight pools of offspring samples. The mRNA was hybridized to a 38K Affymetrix chicken gene expression microarray, and the DNA was used for subsequent tiling array analysis of methylation. Between populations, there were in total 281 significantly (FDR-corrected P < 0.05) differentially expressed (DE) genes in the parents, and 1674 in the offspring. The lower number of DE genes in the parents could possibly be an effect of the lower power of detection given the smaller biological sample size in this generation. Between families within populations, only a few genes were significantly DE, and also DM was less frequent between families (Additional file 2). This indicates that expression and methylation profiles are relatively stable within breeds, but both may have changed considerably during domestication.

### Transgenerational stability of gene expression profiles

**Figure 1:**
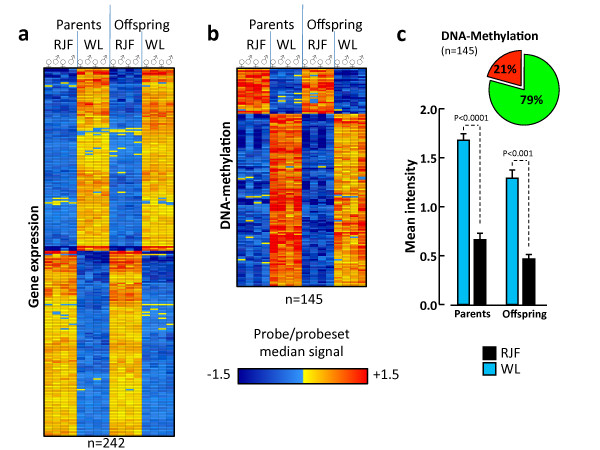
**Gene expression and methylation differences between populations and across generations**. **a**. Heat map, showing the clustering of 242 differentially expressed genes, comparing parental Red Junglefowl (RJF) and White Leghorn (WL) layers, and their offspring. **b**. Heat map, showing clusters of differentially methylated genes comparing parental RJF and WL, and their offspring. Note that the gene set in b is not the same as in a. **c**. Average methylation levels (signal intensity from the microarray) for RJF and WL in the 145 promoter regions included in the heatmap in panel b. Circle-diagram displays the percentage of the promoter regions which were hypermethylated (green) and hypomethylated (red) in White Leghorns.

**Figure 2:**
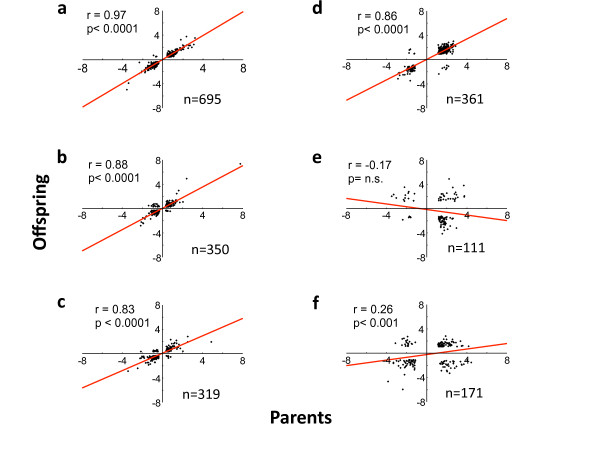
**Correlations of differential expression and methylation of genes between generations**. **a-c**, Correlations between generations of differential gene expression, comparing Red Junglefowl and White Leghorn **(a)**; families within Red Junglefowl **(b)**; and families within White Leghorns **(c)**; **d-f**, Correlation between generations of differential methylation, comparing Red Junglefowl and White Leghorn **(d)**; families within Red Junglefowl **(e)**; and families within White Leghorns **(f)**. The genes included in the analyses were selected based on the fact that they simultaneously occurred in both parents and offspring among top 1000 DE or DM, sorted by fold change.

Out of the significantly DE genes in the parents (comparing populations), 86% percent (n = 242) were also significantly DE in the offspring (Additional file 3), and there was a distinct similarity in the expression differences in both generations (Figure 1a). The overall pattern of fold-change levels between populations (regardless of whether they were significant) was strongly correlated over generations (Figure 2 a), further showing a transgenerational stability in gene expression profiles. Also within populations, the overall pattern of fold-change levels between families was highly correlated across generations (Figure 2 b-c).

We further used signalling intensities of individual probesets on each microarray to correlate global expression levels between parents and their own offspring, compared to offspring of other birds, and found a significantly higher correlation within families than between (mean difference in correlation coefficients 0.0017 ± 0.0002 (SEM), t = 8.2, P < 0.001). This was true both for RJF and WL, and further supports that specific brain gene expression profiles are indeed inherited.

### Gene methylation: inheritance and differences between populations

For analysis of differential methylation (DM), we selected 3623 genes from the list of genes which had the highest fold changes in DE in both generations, both in the between- and within-population comparison. Note that only 281 of these were significant in parents and 1674 in offspring. For each of these genes, 50-75 bp-probes representing a region spanning from -7.25 kb upstream to +3.25 kb downstream of the transcription start point (hence mostly covering promoter regions and other cis-acting regulatory elements) were placed on a custom made tiling array. Methylated DNA immune precipitation (MeDIP) was used to enrich methylated DNA fragments, and after labelling and hybridisation, the relative levels of methylated to un-methylated DNA was assessed for each probe.

Out of the 3623 selected genes, 239 were significantly DM (FDR-corrected P < 0.05) when comparing RJF and WL parents, and 821 were DM in the corresponding comparison in the offspring. A smaller number were classified as DM when comparing between families within population (Table S2). A heat map of the genes classified as DM in both generations showed a highly consistent pattern across generations (Figure 1b). Furthermore, DM levels were significantly correlated between generations when comparing RJF with WL (Figure 2 d), and also to a lesser degree when comparing WL, but not RJF families (Figure 2 e-f).

Of the 145 genes which were significantly DM in both generations (Additional file 4; Additional file 5), 79% were hypermethylated in WL (Figure 1c). This is a highly significant bias (χ^2 ^= 49.8, P < 0.0001), indicating that this breed has acquired novel methylation patterns during its selection history.

We further analysed the relationship between DM and DE on the 3623 selected genes. There was no overall correlation between the level of DM of a gene (% of DM probes) and the degree of DE of the same gene (Additional file 4). Furthermore, there was no overrepresentation of DE genes among the top 100 DM promoters when compared to a random sample of 100 DM genes (χ^2 ^= 2.1, P > 0.05). This is contrary to the common notion that methylation causes down-regulation of gene expression, but similar findings have recently been reported from other species, for example humans [16,17]. The finding is quite surprising, and indicates that the specific sites of methylation may be of major importance for gene regulation. For example, there may be a substantial difference between methylation of transcription factors compared to insulator sequences. Since we only analysed a 10 kb region around the transcription start site of each gene, we can not exclude that DM in other, more distant regulatory regions may be more closely connected to the expression level.

**Figure 3:**
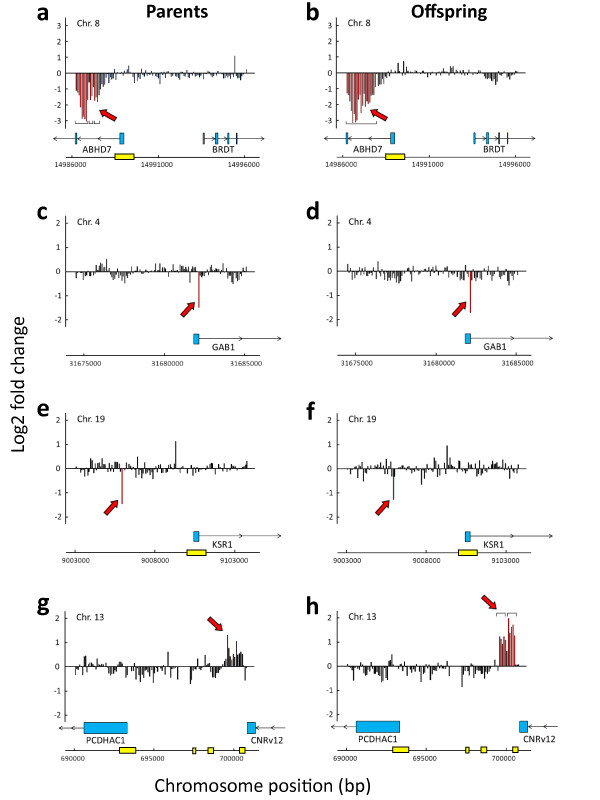
**Transgenerational stability of methylation patterns in specific genes**. **a-h**, Differential methylation levels (Log2 fold change) of promoter regions, comparing Red Junglefowl and White Leghorn, are shown with a resolution of 50-75 bp-regions in parents and offspring for each of the genes **a, b**, *ABHD7*, **c, d**, *GAB1*, **e, f**, *KSR1*, and **g, h**, *RAPGEF1*. Transcription direction and exons (blue boxes) are shown, and red arrows point at bars or groups of bars where significant levels of differential methylation are found (also indicated by red bars). Locations of CpG-islands are indicated in yellow for each region.

To illustrate examples of the transgenerationally stable methylation patterns observed, we show methylation graphs for four genes (*ABHD7, GAB1, KSR1 *and *PCDHAC1*) in Figure 3. In all four, the methylation pattern was reliably inherited, shown by the fact that the DM pattern was highly similar in parents and offspring. *ABHD7 *showed extensive DM ranging several kb downstream of the transcription start site. In none of the four genes, the significantly DM loci were in CpG-islands, so methylation must have targeted cytosines in other genomic contexts. Extensive methylation of non-CpG regions have recently also been reported for the human methylome [16,17], and it remains unknown which functions these epigenetic variants may serve.

### Verification of differential methylation with independent animals, tissues and method

To verify the results of the array-based methylation analysis, we arbitrarily selected four genes, which were DM on the tiling arrays in either parents or offspring, *FUCA1, PCDHAC1, TXNDC16*, and *RUFY3*, and replicated the findings for those, using a different technique and a different animal material. Hypothalamus/thalamus regions from eight five-weeks old RJF and eight WL (same strains as earlier, but different parents) were dissected and treated as described above. The DNA was bisulfite-treated, and the degree of methylation was determined in the regions that were significantly DM on the tiling array using methylation sensitive high resolution melting (MS-HRM) analysis.

**Figure 4:**
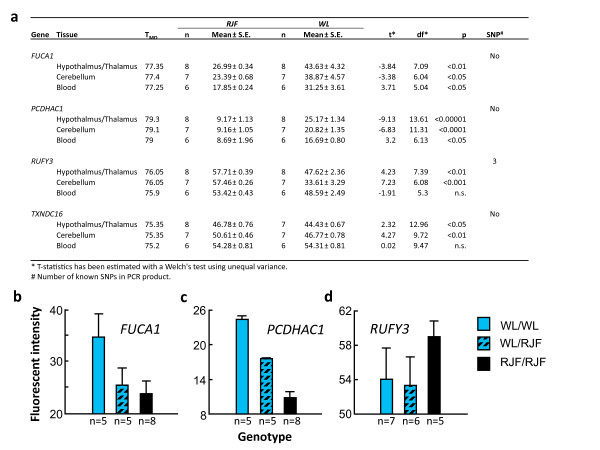
**Verification of differential methylation of four arbitrarily chosen genes**. **a**. Methylation differences between Red Junglefowl and White Leghorn in three different tissues, estimated by methylation sensitive high resolution melting (MS-HRM) analysis. The table shows the normalized fluorescent intensities together with the statistical analysis of 6-8 individual samples per population, at a temperature where positive and negative methylation controls showed highest intensity difference (T_md_). The right column shows the number of SNPs present in the PCR-product amplified by the primers for the region analysed. **b-d**, The average methylation level of the promoter regions of three of the genes, *FUCA1 ***(b)**, *PCDHAC1 ***(c) **and *RUFY3 ***(d)**, in blood samples from F8-generation intercross birds between Red Junglefowl and White Leghorns. The birds differed as indicated in their genotype on an SNP-marker close to the differentially methylated locus.

All four genes were significantly DM in the same direction as found on the tiling array (*FUCA1 *and *PCDHAC1 *hypermethylated in WL; *RUFY3 *and *TXNDC16 *hypomethylated) (Figure 4a). This suggests that the tiling array produced reliable results and that the observed methylation differences are representative for the population differences at large.

In order to check for tissue-specificity of the DM, we also performed HRM on the same four genes, using DNA-pools prepared from cerebellum and blood from the offspring samples included in the tiling arrays. All four genes were significantly DM in cerebellum. In blood, *FUCA1 *and *PCDHAC1 *were significant, while *RUFY3 *showed a tendency for DM (P = 0.08) (Figure 4 a). The fact that *TXNDC16 *was not DM in blood indicates that this gene shows tissue-specific, heritable methylation.

### Genetic stability of methylation differences

There is a risk that the methylation differences detected by the MeDIP technique could be affected by sequence differences in the promoter regions used for the arrays. To exclude this possibility, we used the recently published resequencing data of Red Junglefowl and domestic chickens [18] to check the 145 significantly DM probes in both parents and offspring for possible deletions, insertions and SNP density. Apart from occasional SNPs (Additional file 5), no major sequence differences were detected.

The methylation differences observed may be a result of either inheritance of the epigenetic changes independently of genetic changes, or result from sequence differences which secondarily affect methylation at close or remote loci. This is more difficult to differentiate, since it would require extensive resequencing data of the individuals actually used in the study, combined with, for example, methylation QTL-studies.

To suggestively analyse whether differential methylation of specific loci are caused by sequence differences we decided to study its genetic stability and segregation over several generations. For this purpose, we used a total of 18 birds from the eighth generation of an intercross between RJF and WL. In this population, genetic recombinations in each generation have broken up the linkage between adjacent loci, and we could therefore check for both stability of the methylation sites, and for possible cis- or trans-regulation of these.

From this group of advanced intercross birds, we selected individuals, which were homozygous for either the WL or RJF-allele, or heterozygotes, of an SNP located within 176-1449 kb of the locus showing DM. Using HRM analysis on DNA from blood, extracted from these different genotypes, we again analysed the methylation on *FUCA1, PCDHAC1 *and *RUFY3 *in these individuals (Figure 4b). For *FUCA1*, we found two different non-significant, but distinct, methylation levels, where the birds homozygous for the WL-marker were hypermethylated, and heterozygotes were similar to the ones homozygous for the RJF-marker (P = 0.07). With respect to *PCDHAC1*, the three genotypes were significantly different (P < 0.001), with heterozygotes having a methylation level falling between the hypermethylated WL homozygotes, and the RJF genotypes. *RUFY3 *showed a high level of methylation, which was not significantly different between the three genotypes. Hence, two of the three DM loci were stable over the eight generations of intercrossing, and tended to segregate according to genotype at the locus. This is consistent with a cis-regulating mechanism, showing a dominant inheritance of hypomethylation in genotypes with RJF alleles for *FUCA1*, and an intermediate, codominant inheritance in *PCDHAC1. RUFY3 *may possibly be under control of trans-acting loci, which have segregated during the intercrossing.

Although these results are not conclusive, they suggest that sequence differences may determine the DM for at least two of the three loci, possibly for all of them. This further suggests that selection during domestication may have targeted genotypes which modify the epigenomes, perhaps affecting phenotypes indirectly.

### Genetic pathways

To examine which genetic pathways and functions that may have been affected by DE and DM, we performed a gene ontology (GO) analysis. We analysed the DM and the DE genes in each generation separately, and then selected those GO-terms and KEGG pathways (P < 0.1), which were significantly enriched in both generations (Additional file 6).

A majority of the enriched GO terms are related to phosphorylation and kinase activity, important aspects of intercellular signalling. Looking specifically at the KEGG pathways enriched among DM and DE genes in offspring only (where the biological sample is considerably larger), the analysis shows that MAPK signalling pathway (which, for example, is associated with stress responses), long-term potentiation (affecting memory consolidation), neurotrophin signalling pathway (involved in neural differentiation) and GnRH signalling pathways (related to reproduction) are enriched. All these are potentially interesting from a domestication perspective, in that they may be related to well documented differences between RJF and WL in stress tolerance, behaviour and reproduction.

### Over-representation of epigenetically affected genes in selective sweep regions

We considered that the epigenetic differences between the layer breed and their ancestor could reflect general effects of selection during domestication, as suggested above, perhaps being related to differences in the domestication induced phenotypes, such as growth, feeding behaviour and social tolerance. If so, we would expect the epigenetic differences to be accumulated in genomic regions which have been under selection during domestication. Therefore, we compared our data to one of our earlier, and recently published, datasets on chickens [18]. This dataset consists of an extensive list of selective sweeps related to chicken domestication, based on resequencing of populations of RJF and a number of domesticated breeds. In total, 149 selective sweeps present in all domestic chickens, and 134 present in egg laying breeds only, were used. A sweep was defined as a 40 kb region where the heterozygosity Z-score was below -4.

There were 216 DE genes (DE in both generations) with annotated loci within the 975 Mb of the genome covered by the sweep analysis. Five of them were situated within 50 kb of selective sweeps present in all domestic chickens (non-significant association, based on a permutation test; P > 0.1), and nine in the laying breed sweeps (significantly more than expected by chance; P < 0.05).

We performed the same analysis on 134 DM loci, and found that four were within 50 kb of sweeps in all domestic chickens (non-significant; P > 0.1), and six in laying breed sweeps (P < 0.05). The significant overlapping genes in laying breed sweeps are shown in Additional file 7.

It is interesting to note that *ABHD7*, which was the strongest DM and one of the strongest DE genes in our experiment, is positioned in a laying breed sweep. This gene is named *EPHX4 *in humans, and is related to detoxification of exogenous chemicals [19]. Based on its position in a selective sweep, and its differential methylation and expression, it would appear that the epigenetic variant of the gene (or the genotype affecting the epigenetic state of it) may have been selected during domestication. *KSR1*, an important gene in MAPK/Ras dependent signalling [20], as well as *ADRA2C*, an alpha adrenoreceptor that may be related to egg laying [21] and regulation of the sympathetic stress reaction [22], are also situated in laying breed sweeps.

Although our data do not allow us to conclude on which genes and which sweeps that are associated with specific phenotypes, they suggest that selection of epigenetic variation may have been an important part of chicken domestication.

### General discussion

Our findings show that differential methylation and gene expression in hypothalamus/thalamus are abundant in a comparison between domesticated White Leghorn layers and their wild ancestors, the Red Junglefowl. Many of these epigenetic differences are inherited, demonstrating transgenerational stability. It is possible that these differences are a result of selection during domestication, targeting either sequence differences which affect epigenetic states of specific loci, or epigenetic states which are not related to sequence differences.

The causal relationship between methylation and gene regulation is not clear, since differential methylation was associated with both up- and down-regulation of the gene expression, or did not affect it at all. Since similar dissociation between methylation and gene expression has recently been found in the human genome as well [17,23], this indicates that epigenetic regulation is more complex than previously assumed. Whereas it is often believed that methylation of promoter regions is associated with down-regulation of gene expression, our results indicate that gene regulation is more complex than so. For example, chromatin structure may be more important than commonly assumed. Furthermore, we found that CpG-islands are not always methylated, so there may also be evolutionary contraints on methylation sites, hence affecting the rate with which epigenetic adaptations may occur in different parts of the genome. Although speculative, these issues should be considered in future research.

Some of the methylation differences observed appear to be tissue-specific, whereas others affect a wider range of cells. The mechanism whereby differential methylation at a particular locus only in, for example, the brain can be transferred from parents to offspring remains elusive. In *Drosophila*, similar observations have been made with respect to gene expression, where induced differences specifically in the brain can be transmitted via sperm and cause tissue specific effects in the next generation [24]. Possibly, microRNA regulation may be involved [25], and both sperm and eggs may also transfer specific histone variants [26]. There is also a close link between genetics and epigenetics, in that the epigenetic state of a particular locus is determined by both genetic and epigenetic variations at other loci [23,25].

Stable inheritance of epigenetic variants has been demonstrated in plants [7,13,27,28]. Also in mammals (mainly in rodents and humans), there is increasing evidence that this occurs widely [10,29]. Our results are the first to demonstrate the same in birds, and furthermore show a long-term stability over several generations of specific methylation states.

Although we have only studied one population each of Red Junglefowl and domesticated chickens, the observations in this experiment could indicate that selection of favourable epigenomes, or genotypes favourably affecting the epigenome, may have been an important aspect of chicken domestication. However, further studies are needed, where methylation of specific genes are analysed in a wide range of domesticated populations, analogous to the recent study of sequence variation in the domesticated chicken genome [18].

## Conclusions

Both gene expression and promoter methylation profiles in hypothalamus differed between White Leghorn chickens and their wild ancestors, the Red Junglefowl. Family differences within breed as well as breed differences were maintained in the offspring, showing a reliable inheritance of epigenetic states, which may have been an important mechanism involved in the rapid evolutionary changes of chickens during domestication.

## Methods

### Ethics statement

The experiment and all its procedures were approved by the regional Ethical Committee.

### Animals and selection

We used two breeds of chicken: a domesticated egg layer White Leghorn (WL) and a wild type Red junglefowl (RJF). The population backgrounds have been described elsewhere [30]. The WL was an outbred mixture of different leghorn breeds, established 1970, and since then kept in a closed population at the university. The RJF stem from an outbred zoo population, kept at the university for 10 years (one generation per year) with a generation size of about 100 individuals. For more details about breeding and housing routines, see [6].

From each breed, two breeding pairs were selected based on divergent performance in four different fear tests conducted between 153-168 days of age. The selection criterion in each test was the frequency of standing/sitting alert, where a longer duration signifies a higher fear level [6]. The tests were: 1) Behaviour in an open field during 10 min (this test was repeated at 286 days for check of consistency); 2) Behavioural response for 5 min after novel object introduction; 3) Behaviour 5 min before and 5 min after exposure to an aerial predator model; 4) Fearful behaviour toward a human. All tests were conducted in the same arena (0.5 × 1.5 m), and details of the test procedures have been described elsewhere [6]. All birds were weighed at 373 days of age.

Eggs were collected for five weeks from each individual female in the breeding pairs and incubated in the same incubator in three consecutive batches. Numbers of eggs in the batches were balanced for family. Each batch consisted of 9-18 birds, and the batches were housed separately in mixed groups under the same conditions as the parents. At day 20 all offspring were tested in a similar open field arena as the parents.

For HRM verification of the breed differences in methylation levels, we bred 8 offspring of each breed (four females and four males), using different animals (but same populations) as those above. These chicks were culled and sampled in the same way as described below.

For studying the long-term stability of methylated loci, we used 18 birds from generation eight of an intercross between RJF and WL. The details of this intercross has been described elsewhere [3]. Briefly, one male RJF and three WL (same populations as those used in other parts of the present experiment) were used to breed 36 F1, and these were intercrossed to produce about 1000 F2. From generation F3 onwards, about 100 birds per generation have been maintained by random mating and pedigree hatching up to generation F8.

### RNA and DNA isolation

Parents were culled at day 373 after hatch and their offspring at day 21. A sample of six male and six female offspring (balanced between batches) were chosen from each family. A part of the brain enriched of thalamus/hypothalamus was anatomically dissected and immediately snap frozen in liquid nitrogen, and blood was collected immediately after culling. Samples were homogenized in TRI-reagents (Ambion) using the FastPrep^® ^-24 homogenization system with Lyzing matrix D tubes (MP Biomedicals).

RNA was further extracted with the same method as has been used previously [8,9] following the protocol of the TRI-reagent manufacturer, except for a modification adding 0.25 ml isopropanol and 0.25 ml RNA-precipitation solution (1.2 M NaCl, 0.8 M disodium citrate). After the TRI-based RNA extraction, an RNeasy kit (Qiagen) was used to further purify the samples.

DNA was extracted from the same TRI homogenate as the RNA. Precipitation was done on ice by adding 150 μl of 100% ethanol to 300 μl TRI homogenate followed by gentle vortexing and 5 min 4°C incubation. After centrifugation in 12000× g for 10 min, the supernatant was discarded and the pellet resuspended in the ATL buffer of the DNeasy kit (Qiagen). 4 μl of 10 mg/ml Rnase A was then added, incubated 2 min at room temperature, followed by addition of 20 μl of 10 mg/ml proteinase K (Qiagen) and 3 min incubation at 56°C. DNA was then extracted according to the DNeasy protocol for animal tissue. Quality and quantity of both RNA and DNA was measured on a Bioanalyzer^® ^instrument (Agilent Technologies) and NanoDrop^® ^ND-1000 spectrophotometer (Thermo Scientific).

Both RNA and DNA extractions were treated individually for the parents, but as pools of six same-sex samples in the offspring.

### Gene expression microarray

This part of the experiment was performed at Uppsala Array Platform at Uppsala University, Sweden http://www.medsci.uu.se/klinfarm/arrayplatform. A total of 16 GeneChip Chicken Genome Arrays (Affymetrix Inc.) were used to measure the expression of 33457 transcripts. Biotinylated fragmented RNA was prepared for each sample using standard procedures in GeneChip ^® ^3' IVT Express Kit User's manual (Affymetrix Inc., Rev. 1, 2008). This was followed by array hybridization for 16 h in 45°C under constant rotation. Washing and staining was performed in a Fluidics Station 450 and scanned using the GeneChip Scanner 3000 7 G (Affymetrix Inc.).

### Gene expression data analysis

Analysis of the gene expression was performed using the statistical software R http://www.r-project.org with Bioconductor packages http://www.bioconductor.org. Normalization was done with the RMA method [31] and differentially expressed genes were evaluated using fold change in combination with a Bayes moderated t-test [32] adjusted for false discovery rate [33].

Correlation analysis, comparing the fold change of differential expression in parents and offspring, was done using Statistica v 9.1. Cluster analysis and heat maps were performed with the Genesis software v 1.7.5 [34].

### DNA-methylation tiling array design

For methylation analysis, we selected the genes which had the highest fold change of expression in both generations of the breed comparison in the microarray data. From each gene, the promoter regions, defined as 7.25 kb upstream and 3.25 kb downstream of the transcription start site (Ensemble genebuild WASHUC2), were used to design a custom 385 K DNA-methylation tiling array (Roche-NimbleGen). In total 3623 promoter regions were tiled to the array, with 50-75 mer probes and 100 bp median spacing, by the Madison design team at Roche-NimbleGen.

### Methylated DNA Immunoprecipitation (MeDIP)

Protocols of MeDIP with buffer descriptions and general procedures have been published elsewhere [35,36]. Fragmentation of 6 μg thalamus/hypothalamus DNA was performed using a BRANSON sonifier 250 with a 13 mm disruptor horn (101-147-037) and a 3 mm tapered microtip (101-148-062). Samples were diluted with 450 μl 1 × TE in 1.5 ml tubes and sonicated at 10% amplitude by short 0.5 sec pulses (20 in total) with a rest between pulses of 0.5 sec. Fragment lengths of between 300-1000 bp were verified on a Bioanalyzer (Agilent technologies). After sample denaturation 10 min at 95°C, reference samples of 10 μl was taken from each of the original samples and frozen. The remaining samples underwent methylated DNA immunoprecipitation by first diluting them in 1 × TE to 450 μl, adding 51 μl of 10 × IP buffer and 10 μg of 5-meC antibody (Diagenode). Samples were then incubated in 4°C for 2 h on a rotating platform, whereby 50 μl of clean Dynabeads Protein G (Invitrogen) in 1 × IP buffer was added and followed by an identical 2 h incubation. The beads-antibody-antigen complex was washed 3 times by placing the samples on a DynaMag-spin magnet, discarding the supernatant and adding 1 ml of 1 × IP. Complex digestion was done by adding 250 μl of Proteinase K digestion buffer and 5 μl Proteinase K (20 mg/ml), followed by rotation over night in 50°C. DNA was further purified by phenol/chloroform procedures with glycogen/ethanol -80°C precipitation. The pellet were washed in 100% ethanol and resuspended in 60 μl 1 × TE. All samples, references as well as MeDIP's, were then whole genome amplified using the WGA2 kit (Sigma-aldrich) and purified with QIAquick PCR purification kit (Qiagen).

### DNA-methylation tiling array labeling and hybridization

Labeling and hybridization was performed at Roche-NimbleGen service lab at Iceland, Reykjavik, using standard protocols [37]. In short, MeDIP and reference samples were labeled with Cy5 and Cy3 respectively, using the NimbleGen Dual-Color DNA Labeling Kit. The MeDIP-Cy5 and reference-Cy3 samples from each tissue sample were then co-hybridised to the DNA-methylation tiling array using the NimbleGen Hybridization Kit and Hybridization System. After washing with NimbleGen Wash Buffer Kit the slides were scanned by a NimbleGen MS 200 Microarray Scanner.

### Tiling array data analysis

Methylation data analysis was performed using Bioconductor in the open source R statistical software environment [38]. To not loose genome wide methylation differences, the RINGO package [39] was used to preprocess the data within arrays by Tukey's biweight normalization and between arrays with A-quantile normalization. Significantly differentially methylated probes (FDR adjusted P-values) were extracted using the limma package [32]). Since promoters sometimes involved more than one significant probe, in all comparisons with the gene expression data and correlations across generations, only the most significant probe of each promoter was considered. All significant probes that were stable across generations were checked for the occurrence of SNPs using a list of SNPs detected in a multibreed resequencing study recently published^18^.

### Methylation sensitive high resolution melting analysis

Verification of differentially methylated genes, and analysis of differential methylation in alternative tissues and in F8-intercross birds, was done by methylation sensitivehigh resolutions melting (MS-HRM) analysis, principally as described by [40]. If not said otherwise, all procedures followed manufactures recommendations. DNA was prepared from brain tissues as above and from blood using the DNeasy Blood and Tissue kit adjusted for nucleated blood (Qiagen).

Positive control samples were synthesized by in vitro methylation, using a nuclease-free water diluted reaction mix of 16.5 μl, including an all bird pool of 1 μg DNA, 2 μl 10× NEBuffer2, 2 μl SAM (640 μM), 1 μl SssI methylase (4 U/μl) (New England BioLabs Inc.). After 2 h of incubation at 37°C, an additional 2.5 μl SAM was added to each sample, followed by another 2 h incubation and then termination by heating at 65°C for 20 min.

Negative control samples were synthesized by whole genome amplification on the same all bird DNA pool (10-20 ng/μl) as for the positive control using the REPLI-g Mini Kit (Qiagen). The whole volume of amplified negative controls were then mixed with 200 μl Buffer AL and 200 μl ethanol (99%) and purified with the DNeasy Blood and Tissue kit (Qiagen). 1 μg DNA from both individual samples and controls were bisulfite treated using the EpiTect Bisulfite Kit (Qiagen).

PCR and High resolution melting was performed on a Rotor-Gene 6000 thermocycler (Corbett Research). 1 μl of the bisulfite treated samples/controls were prepared in a 10 μl PCR mix using EpiTect HRM PCR Kit (Qiagen). A calibration series was also amplified using a mixture of positive and negative controls at 100%, 75%, 50%, 25% and 0% of methylated DNA. PCR was performed in 45 cycles as follows: denaturation 10 s at 95°C, annealing at 30 s 54-55°C (primer dependent) and extension 10 s at 72°C. MS-HRM was run in the interval of 70°C to 90°C, with a 2 s 0.05°C steps, acquiring fluorescence data at the Rotor-Gene HRM channel. Primer sequences and annealing temperatures can be seen in Additional file 8. All MS-HRM reactions were run in triplicates.

### Annotation and GO analysis

Affy Chicken ID, EntrezGene ID, EnsembleGene ID, WikiGene ID and chromosomal regions were extracted from the Affymetrix annotation file (release 29), and further annotated with Ensemble's BioMart tool [41]. We used DAVID 6.7 http://david.abcc.ncifcrf.gov to extract significantly enriched gene ontology terms and KEGG pathways within our datasets [42,43]. To increase the possible DAVID hits we first extracted the homologous human Ensembl ID of our chicken genes in BioMart [41]. CpG island prediction was performed with EMBOSS CpGPlot [44].

### Analysis of sweep overlaps

219 DE genes and 134 DM promoters (significant in both parents and offspring) fell within the 975 Mb that previously has been searched for selective sweeps^18^. To investigate whether genes or promoters were significantly enriched in sweep regions, 1000 sets of random intervals were generated over the 975 Mb for each analysis, each interval in each set chosen to represent one DE or one DM gene. The number of overlaps between the randomly generated interval and a sweep (within 50 kb of the sweep) was compared to the actual number of real overlaps. A probability of the observed coincidence of less than 5% was taken as a significant association between DM/DE genes and sweeps.

## Competing interests

The authors declare that they have no competing interests.

## Authors' contributions

DN performed the experiments, analysis and lab work, JB and MJ assisted in HRM-analysis, CR performed the sweep analysis, DW performed the F8-breeding and genotyping, LA developed the intercross analysis, and PJ, together with DN, planned and conceptualised the experiment, and wrote the paper. All authors read and approved the final manuscript.

### Accession numbers

The microarray data from this study have been deposited at ArrayExpress and can be accessed using the following accession numbers: 1) Parents gene expression [E-MTAB-644]; 2) Offspring gene expression [E-MTAB644]; 3) Parents DNA-methylation [E-MTAB-648]; 4) Offspring DNA-methylation [E-MTAB-649]; 4) Tiling array design [E-MEXP-2041])

## Supplementary Material

Additional file 1 **Phenotypes of parents and offspring**. The behavioural scores and weight data for the animals in the experiment. Click here for file

Additional file 2 **Nrs of differentially expressed and methylated genes**. Total numbers of significantly differentially expressed genes or methylated promoters (P < 0.05, FDR-corrected) in the different comparisons. Click here for file

Additional file 3 **Differentially expressed genes**. A full list of all genes found to be differentially expressed, comparing breeds, in both generations, including their chromosomal alignment and accession numbers. Click here for file

Additional file 4 **Expression and methylation**. Gene expression differences plotted against promoter methylation differences between WL and RJF offspring. Click here for file

Additional file 5 **Differentially methylated genes**. A full list of all genes, where the promotors were found to be differentially methylated, in both generations. Click here for file

Additional file 6 **Gene function**. Gene ontology and KEGG pathway annotation for the genes which were either differentially expressed or differentially methylated in both generations, comparing between breeds. Click here for file

Additional file 7 **Selective sweep representation**. Genes which were differentially expressed or methylated between breeds in both generations, and significantly overrepresented in selective sweeps associated with domestication. Click here for file

Additional file 8 **Primer structures**. The bisulfate converted primers used in HRM. Click here for file
