# Supplementary material for: Heritable genome-wide variation of gene expression and promoter methylation between wild and domesticated chickens
Source: BMC Genomics. 2012 Feb 4;13:59. doi: 10.1186/1471-2164-13-59 (PMC3297523; doi:10.1186/1471-2164-13-59)
Supplement: Additional file 1 — Phenotypes of parents and offspring. The behavioural scores and weight data for the animals in the experiment. [file 1471-2164-13-59-S1.PDF]

Additional file 1 | Phenotypes of parents and offspring. The table shows the behaviour score as a composite from a series of fear-tests, and the body weights at different ages for the four families included in the experiment.

		Red junglefowl				*White leghorn*			
		Family 1		***Family 2***		***Family 1***		***Family 2***	
Parents	n =	2		2		2		2	
*Behaviour score**		537	(±39)	315	(±58)	456	(±112)	227	(±92)
Adult weight (g)[#]		1036	(±55)	954	(±28)	2119	(±181)	1613	(±154)
Offspring[$]	n =	17		19		20		17	
Behaviour score		467.8	±17.1	477.1	±15.9	488.5	±19.7 [a]	408.6	±34.5 [a]
Hatch weight (g)		32.4	±0.4 [b]	27.7	±0.3 [b]	49.9	±0.7 [c]	44.4	±0.5 [c]
Weight 3 weeks (g)		109.3	±2.7[d]	93.3	±2.7[d]	177.3	±4.2 [e]	140.9	±2.9 [e]

* Mean score with ± SEM based on inactive behaviour of five fear test (two open fields, novel object, aerial predator and frear for human).

\# Mean weight ± SEM (min and max); min = mother and max = father.

$ Means with ± SEM on one measurement/test

a Significant difference p<0.05 between family within breed (student t-test)

b-e Significant difference p<0.001 between family within breed (student t-test)
