# Supplementary material for: Heritable genome-wide variation of gene expression and promoter methylation between wild and domesticated chickens
Source: BMC Genomics. 2012 Feb 4;13:59. doi: 10.1186/1471-2164-13-59 (PMC3297523; doi:10.1186/1471-2164-13-59)
Supplement: Additional file 2 — Nrs of differentially expressed and methylated genes. Total numbers of significantly differentially expressed genes or methylated promoters (P < 0.05, FDR-corrected) in the different comparisons. [file 1471-2164-13-59-S2.PDF]

Additional file 2| Numbers of significant significantly differentially expressed genes or methylated promoters (P<0.05, FDR-corrected) in the different comparisons. Overlapping genes were differentially expressed or methylated in both generations.

	Between breeds	***RJF Between families***	***WL Between families***
**Gene expression**			
Parents	281	1	1
Offspring	1674	3	4
Overlap	242	1	1
**DNA-Methylation**			
Parents	239	0	15
Offspring	821	124	42
Overlap	145	0	2
