# Supplementary material for: Heritable genome-wide variation of gene expression and promoter methylation between wild and domesticated chickens
Source: BMC Genomics. 2012 Feb 4;13:59. doi: 10.1186/1471-2164-13-59 (PMC3297523; doi:10.1186/1471-2164-13-59)
Supplement: Additional file 3 — Differentially expressed genes. A full list of all genes found to be differentially expressed, comparing breeds, in both generations, including their chromosomal alignment and accession numbers. [file 1471-2164-13-59-S3.PDF]

Additional file 3 | Differentially expressed genes, comparing breeds, in both generations. The table gives the Affymetrix and Ensembl ID and the name of the gene, where available. For each gene the best genomic alignment is shown, and the fold change (FC; negative sign shows lower value in domestic birds) together with the FDR corrected P-value (adj.P).

Identifiers			*Best genomic alignment*			*Parents**		*Offspring**	
**Affymetrix ID**	**Name**	**Ensembl Gene ID**	**Chromosome**	**Start**	**Stop**	**FC**	**adj. P**	**FC**	**adj. P**
Gga.10303.1.S1_at	CAV2	ENSGALG00000009396	1	26874816	26883270	-0.60	<0,05	-0.46	<0.01
GgaAffx.11088.1.S1_at	CCDC91	ENSGALG00000017293	1	74913131	74915796	0.97	<0,05	0.41	<0.01
Gga.8353.1.S1_at	CSTB	ENSGALG00000014410	1	79861286	79865657	0.53	<0,01	0.47	<0.001
Gga.5413.1.S1_at	DNAJC15	ENSGALG00000016965	1	171000402	171025711	-0.51	<0,05	-0.35	<0.05
GgaAffx.10933.1.S1_s_at	FRY	ENSGALG00000017075	1	178972178	178997683	0.72	<0,05	0.43	<0.05
Gga.3151.1.S1_at	IFNAR2	ENSGALG00000015938	1	108690429	108702476	0.52	<0,05	0.55	<0.01
Gga.17504.1.S1_at	ILDR2	ENSGALG00000015458	1	95210572	95213044	-0.74	<0,01	-0.67	<0.01
Gga.5671.1.S1_at	KRAS	ENSGALG00000014033	1	69356187	69357771	-0.93	<0,01	-0.80	<0.001
GgaAffx.11956.1.S1_at	LIMS1	ENSGALG00000016807	1	140494229	140593771	-1.23	<0,01	-1.07	<0.01
Gga.1247.1.S1_at	LOC396098	ENSGALG00000015461	1	95262560	95274198	1.26	<0,05	0.91	<0.001
Gga.2280.1.S1_at	LOC418607	ENSGALG00000016418	1	123401432	123417585	-0.67	<0,05	-0.64	<0.01
GgaAffx.10777.1.S1_at	LOC418747	-	1	141847270	141970676	0.42	<0,05	0.40	<0.01
GgaAffx.25206.4.S1_s_at	LOC419011	ENSGALG00000017234	1	191872775	191933212	0.59	<0,01	0.57	<0.05
Gga.10770.2.S1_a_at	LOC425324	-	1	140575727	140632477	-0.84	<0,05	-0.94	<0.001
Gga.10770.1.S1_x_at	LOC425324	-	1	140609697	140612288	-0.94	<0,05	-1.08	<0.001
Gga.7035.1.S1_x_at	LOC769722	ENSGALG00000019142	1	136429230	136431841	-0.53	<0,01	-0.51	<0.001
Gga.10770.2.S1_x_at	LOC771168	-	1	140575727	140632477	-1.15	<0,05	-1.02	<0.001
GgaAffx.12879.1.S1_s_at	MAPK11	ENSGALG00000008612	1	21806995	21828380	-0.37	<0,05	-0.33	<0.01
Gga.12769.1.S1_s_at	NHP2L1	ENSGALG00000011931	1	51431254	51434776	1.07	<0,05	0.96	<0.001
GgaAffx.20202.1.S1_at	PION	ENSGALG00000008312	1	13688829	13690014	0.50	<0,01	0.46	<0.05

GgaAffx.25213.1.S1_at	RAB30	ENSGALG00000017248	1	193426339	193437936	1.12	<0,01	1.16	<0.001
Gga.15973.1.S1_at	RIPK4	ENSGALG00000016145	1	112469983	112471602	1.32	<0,05	1.16	<0.001
Gga.13575.1.S1_at	RPL21	ENSGALG00000017100	1	180611224	180614383	0.53	<0,05	0.56	<0.01
Gga.4827.1.S2_at	SLC25A6	ENSGALG00000016691	1	133256810	133261425	0.63	<0,05	0.57	<0.01
Gga.9636.2.S1_a_at	SNORA32	ENSGALG00000017220	1	190093079	190095498	1.09	<0,05	0.88	<0.01
Gga.12674.2.A1_a_at	TMEM131	ENSGALG00000016751	1	136298151	136298927	0.64	<0,05	0.70	<0.001
GgaAffx.12143.1.S1_s_at	TRAPPC2	ENSGALG00000016578	1	126388986	126394653	-1.06	<0,001	-1.06	<0.0001
GgaAffx.8978.2.S1_s_at	TTLL1	ENSGALG00000014163	1	70649010	70665014	0.74	<0,05	0.56	<0.01
GgaAffx.20887.1.S1_at	ZYX	ENSGALG00000014688	1	80670681	80671502	-1.69	<0,01	-1.57	<0.01
Gga.13202.1.S1_at	-	-	1	148560531	148563744	-1.51	<0,01	-1.58	<0.0001
Gga.15146.1.S1_at	-	-	1	83426683	83427810	0.43	<0,05	0.28	<0.05
Gga.16327.1.S1_at	-	-	1	61980413	61982103	0.55	<0,05	0.60	<0.001
Gga.17333.1.S1_at	-	ENSGALG00000015458	1	95217192	95218450	-0.72	<0,05	-0.63	<0.001
Gga.3070.1.S1_at	-	-	1	138026773	138031765	0.83	<0,05	0.69	<0.01
GgaAffx.20473.1.S1_at	-	-	1	108118671	108120114	0.79	<0,01	1.00	<0.01
GgaAffx.20483.1.S1_at	-	-	1	106519751	106521879	-0.53	<0,05	-0.23	<0.05
GgaAffx.20959.1.S1_at	-	-	1	140485303	140486560	-0.78	<0,05	-0.59	<0.01
GgaAffx.10312.1.S1_s_at	BAI1	ENSGALG00000016160	2	153292043	153496519	0.58	<0,05	0.45	<0.05
GgaAffx.23472.1.S1_at	CHN2	ENSGALG00000011164	2	33567958	33728254	-0.78	<0,05	-0.66	<0.05
GgaAffx.23727.1.S1_s_at	CLASP2	ENSGALG00000011995	2	44703896	44837642	-0.37	<0,05	-0.27	<0.05
Gga.16343.1.S1_at	COL14A1	ENSGALG00000016411	2	142375868	142391504	0.70	<0,05	1.21	<0.01
GgaAffx.4144.1.S1_s_at	DIP2C	ENSGALG00000006652	2	9863350	9995631	0.76	<0,01	0.65	<0.01
Gga.2304.1.S1_at	EGFR	ENSGALG00000012363	2	51962154	51985944	0.70	<0,05	0.53	<0.05
GgaAffx.23916.1.S1_at	HUS1	ENSGALG00000012450	2	56437561	56444515	-0.52	<0,05	-0.32	<0.05
Gga.15024.1.A1_at	IL6	ENSGALG00000010915	2	30896230	30896930	0.67	<0,05	1.11	<0.001
GgaAffx.22012.1.S1_at	LARP5	ENSGALG00000006672	2	10209758	10240084	-1.26	<0,01	-1.37	<0.01
Gga.19413.1.S1_at	LOC426058	-	2	137795868	137797694	-0.72	<0,05	-0.91	<0.01

Gga.18976.1.S1_at	LOC431452	ENSGALG00000016072	2	134850506	134854458	0.88	<0,01	0.88	<0.001
Gga.1454.1.S1_at	LSM5	ENSGALG00000012209	2	48174130	48179357	0.73	<0,05	0.82	<0.01
Gga.5888.2.S1_a_at	MLL3	ENSGALG00000006264	2	6484781	6486027	1.02	<0,01	0.92	<0.01
Gga.2854.1.S1_at	MRPL32	ENSGALG00000012337	2	51293970	51296442	1.07	<0,05	1.16	<0.001
GgaAffx.7188.3.S1_s_at	NGLY1	ENSGALG00000011304	2	37909795	37925390	-0.96	<0,05	-0.98	<0.001
Gga.17001.1.S1_at	OXR1	ENSGALG00000016079	2	136686632	136754261	-0.51	<0,05	-0.34	<0.05
GgaAffx.22642.1.S1_s_at	SLC39A12	ENSGALG00000008602	2	19299289	19333929	-0.42	<0,05	-0.41	<0.01
GgaAffx.7823.1.S1_s_at	STK17A	ENSGALG00000012351	2	51638014	51664930	0.59	<0,01	0.67	<0.001
Gga.1331.2.S1_a_at	TOP2B	ENSGALG00000011300	2	37869656	37873527	1.58	<0,01	1.54	<0.001
Gga.12854.1.S1_at	TPK1	ENSGALG00000012369	2	53892385	54125016	-0.60	<0,05	-0.72	<0.01
GgaAffx.11392.1.S1_s_at	UBE3C	ENSGALG00000006461	2	8533447	8597209	-0.52	<0,05	-0.46	<0.01
Gga.13841.1.S1_at	-	-	2	151279936	151281349	0.35	<0,05	0.39	<0.05
Gga.15024.1.S1_at	-	-	2	30896230	30896930	1.70	<0,01	1.90	<0.001
Gga.15291.1.S1_at	-	-	2	137578537	137579856	-0.42	<0,05	-0.41	<0.01
Gga.16108.1.S1_at	-	-	2	9811691	9813476	2.15	<0,01	2.19	<0.0001
Gga.17236.1.S1_x_at	-	-	2	145485057	145486315	0.87	<0,01	0.95	<0.01
Gga.18034.1.S1_at	AHCTF1P	ENSGALG00000025394	3	35028494	35038511	-0.61	<0,05	-0.58	<0.05
GgaAffx.24308.1.S1_s_at	BCLAF1	ENSGALG00000019967	3	57145009	57175786	0.51	<0,05	0.43	<0.05
Gga.6127.2.S1_a_at	CABC1	ENSGALG00000009082	3	13350506	13354215	0.98	<0,01	0.93	<0.05
Gga.19042.1.S1_s_at	CDC5L	ENSGALG00000016704	3	111666366	111674778	0.73	<0,05	0.58	<0.05
GgaAffx.20193.1.S1_at	DYNC2LI1	ENSGALG00000009954	3	26388445	26398825	-0.92	<0,05	-0.85	<0.01
Gga.2688.1.S1_s_at	ELP3	ENSGALG00000016624	3	108485803	108490882	-0.42	<0,05	-0.43	<0.01
GgaAffx.8875.1.S1_at	GNG4	ENSGALG00000010986	3	39606113	39611879	0.80	<0,05	0.64	<0.05
GgaAffx.9460.1.S1_at	GOPC	ENSGALG00000014902	3	66017662	66042221	-0.73	<0,05	-0.67	<0.001
Gga.18737.1.S1_at	GPCPD1	ENSGALG00000009130	3	17876584	17877292	3.18	<0,0001	3.50	<0.0001
Gga.19526.1.S1_at	LOC421238	-	3	3560156	3566211	0.70	<0,05	0.81	<0.01
Gga.14381.1.S1_a_at	LOC769848	ENSGALG00000018111	3	47787362	47789645	1.28	<0,05	1.46	<0.01

Gga.6121.1.S1_at	LRRN4	ENSGALG00000008815	3	16237220	16239309	-0.92	<0,05	-0.48	<0.05
Gga.15726.1.S1_at	MTRF1L	ENSGALG00000013609	3	51508598	51523106	-0.84	<0,05	-0.72	<0.01
GgaAffx.11373.1.S1_s_at	OSTM1	ENSGALG00000015306	3	70177176	70188138	-0.42	<0,05	-0.36	<0.01
GgaAffx.12172.1.S1_s_at	RCJMB04_10c4	ENSGALG00000009981	3	26686984	26704143	-0.46	<0,05	-0.64	<0.05
GgaAffx.23305.1.S1_s_at	SDCCAG8	ENSGALG00000010713	3	36387444	36485269	0.43	<0,05	0.60	<0.01
Gga.6981.1.S1_at	SF3B5	ENSGALG00000020000	3	47746971	47748152	-1.61	<0,05	-1.45	<0.01
GgaAffx.5539.1.S1_s_at	SLC1A4	ENSGALG00000008811	3	9840436	9864172	0.50	<0,05	0.24	<0.05
Gga.7876.1.S1_at	TTC27	ENSGALG00000010538	3	32610034	32712377	-0.41	<0,05	-0.28	<0.05
Gga.13283.1.S1_at	TXNDC13	ENSGALG00000008853	3	15552714	15554373	0.61	<0,01	0.59	<0.01
Gga.12416.1.A1_at	-	-	3	109211513	109219460	-0.76	<0,01	-0.65	<0.01
Gga.15556.1.S1_at	-	-	3	23019250	23062695	-1.76	<0,05	-1.94	<0.001
Gga.18012.1.S1_at	-	-	3	33350362	33351680	1.36	<0,01	1.27	<0.0001
Gga.7370.1.S1_at	-	-	3	68647858	68648927	-0.61	<0,01	-0.61	<0.001
GgaAffx.21584.1.S1_at	-	ENSGALG00000014883	3	65461633	65462438	-0.71	<0,05	-0.93	<0.05
Gga.12964.1.S1_at	C4orf16	ENSGALG00000012079	4	58747511	58749453	-0.35	<0,05	-0.19	<0.05
Gga.5494.1.S1_at	CPE	ENSGALG00000009563	4	24980967	25028502	1.06	<0,05	0.78	<0.01
GgaAffx.23098.3.S1_s_at	DCLK2	ENSGALG00000010040	4	33931061	33963572	-0.49	<0,05	-0.35	<0.05
Gga.16413.3.S1_at	FGFR3	ENSGALG00000015708	4	86424743	86430878	0.44	<0,05	0.28	<0.05
Gga.10005.1.S1_at	G3BP2	ENSGALG00000010882	4	46251010	46265510	-1.42	<0,01	-1.75	<0.0001
Gga.19508.1.S1_s_at	GAB1	ENSGALG00000009898	4	31661445	31698900	0.83	<0,05	0.46	<0.01
Gga.16916.2.S1_at	GLA	ENSGALG00000004948	4	1999427	2007975	0.57	<0,05	0.55	<0.01
GgaAffx.6806.1.S1_at	GLRA3	ENSGALG00000010781	4	45308909	45381521	-0.41	<0,05	-0.37	<0.01
Gga.8224.1.S1_at	LOC422694	-	4	58780811	58781799	-0.81	<0,01	-0.55	<0.001
Gga.11794.1.S1_s_at	OTOP1	ENSGALG00000014987	4	81464209	81465751	-0.93	<0,05	-0.54	<0.05
GgaAffx.12585.1.S1_at	RCJMB04_18k24	ENSGALG00000010105	4	35021822	35040706	1.36	<0,01	1.45	<0.001
Gga.4516.2.S1_s_at	SEP11	ENSGALG00000011476	4	51155693	51175605	-0.59	<0,05	-0.43	<0.05
Gga.2035.1.S1_at	SLC10A7	ENSGALG00000010006	4	32522077	32523103	0.49	<0,05	0.64	<0.01

Gga.19825.1.S1_at	SLC25A43	ENSGALG00000008682	4	16695862	16698829	-0.75	<0,05	-0.62	<0.01
GgaAffx.23669.2.S1_s_at	YTHDC1	ENSGALG00000011795	4	53261261	53282637	0.53	<0,05	0.47	<0.05
Gga.18546.1.S1_at	-	-	4	8782248	8783428	0.87	<0,05	0.88	<0.01
Gga.5462.1.S1_at	-	-	4	11009148	11010279	-0.51	<0,05	-0.42	<0.01
Gga.4647.1.S1_at	ACYP1	ENSGALG00000010307	5	40563084	40564044	0.86	<0,01	0.81	<0.001
GgaAffx.12611.1.S1_at	ANGEL1	ENSGALG00000010372	5	41300801	41309445	-0.47	<0,05	-0.51	<0.01
GgaAffx.6269.1.S1_at	C14orf126	ENSGALG00000009978	5	36841729	36849022	0.93	<0,05	0.86	<0.001
Gga.12562.2.S1_a_at	C14orf153	ENSGALG00000011526	5	52924502	52932512	-0.88	<0,05	-0.71	<0.01
Gga.16585.1.S1_at	C15orf41	ENSGALG00000009801	5	33772174	33887960	-0.54	<0,05	-0.34	<0.01
GgaAffx.22101.1.S1_s_at	CHKA	ENSGALG00000006917	5	17303062	17316271	-0.80	<0,05	-0.82	<0.01
Gga.6539.1.S1_at	LOC423593		5	61318668	61324276	-1.18	<0,01	-1.22	<0.001
GgaAffx.5419.1.S1_at	LOC771361	ENSGALG00000008599	5	26810571	26811360	-0.95	<0,01	-1.23	<0.001
Gga.6099.1.S1_at	LOC771753	ENSGALG00000009292	5	28349064	28354253	0.52	<0,05	0.50	<0.05
GgaAffx.23778.1.S1_s_at	NAT12	ENSGALG00000012098	5	58092887	58108912	-1.22	<0,01	-1.24	<0.001
GgaAffx.23745.1.S1_s_at	QSER1	ENSGALG00000012030	5	5690402	5705749	0.84	<0,05	0.55	<0.01
Gga.349.1.S1_at	RGS6	ENSGALG00000009368	5	28880141	28906232	-0.40	<0,05	-0.32	<0.01
Gga.1686.1.S1_s_at	TGFB3	ENSGALG00000010346	5	40870865	40879234	0.52	<0,05	0.33	<0.01
Gga.13241.1.S1_at	-	-	5	47973486	47974423	-0.54	<0,05	-0.46	<0.001
Gga.14306.1.S1_at	-	-	5	52320758	52357046	0.82	<0,01	0.90	<0.01
Gga.18320.1.S1_at	-	-	5	30014168	30014880	0.54	<0,05	0.65	<0.01
GgaAffx.20610.1.S1_at	-	-	5	43301280	43302267	0.46	<0,05	0.42	<0.01
Gga.12309.1.S1_at	AIFM2	ENSGALG00000004777	6	13065208	13070209	0.67	<0,01	0.64	<0.01
Gga.5161.2.S1_s_at	ANK3	ENSGALG00000003135	6	10136981	10138448	1.11	<0,05	1.10	<0.01
GgaAffx.5983.1.S1_at	ATE1	ENSGALG00000009502	6	32510301	32579168	-0.91	<0,05	-0.77	<0.001
Gga.12432.1.S1_at	C10orf57	ENSGALG00000021231	6	6180970	6186092	0.78	<0,01	0.54	<0.001
Gga.588.1.S1_at	GFRA1	ENSGALG00000009173	6	30088280	30225382	2.59	<0,05	2.26	<0.01
GgaAffx.4033.1.S1_at	HTR7	ENSGALG00000006478	6	20683255	20704150	-0.80	<0,01	-0.97	<0.0001

GgaAffx.25692.2.S1_s_at	JMJD1C	ENSGALG00000002942	6	9039460	9068965	-0.65	<0,01	-0.75	<0.001
Gga.4452.1.S1_at	NRBF2	ENSGALG00000002959	6	9076994	9079283	-0.71	<0,05	-0.54	<0.05
GgaAffx.1515.3.S1_s_at	OGDHL	ENSGALG00000002266	6	3877412	3915904	-1.12	<0,05	-1.05	<0.0001
GgaAffx.11939.1.S1_at	POLR3A	ENSGALG00000004947	6	14217315	14248769	0.42	<0,05	0.36	<0.01
Gga.9826.1.S1_at	RPP30	ENSGALG00000006486	6	20711742	20728322	1.06	<0,05	0.71	<0.05
Gga.763.1.S1_at	TECTB	ENSGALG00000008802	6	28134553	28142630	0.53	<0,05	0.62	<0.01
GgaAffx.20932.1.S1_s_at	ZNF511	ENSGALG00000003533	6	10488132	10490606	-1.79	<0,01	-2.18	<0.0001
Gga.11128.1.S1_at	-	-	6	12378835	12379450	0.90	<0,01	0.97	<0.001
Gga.11206.1.S1_at	-	-	6	7727522	7728510	0.79	<0,01	0.54	<0.01
Gga.8540.1.S1_at	-	-	6	9797411	9798380	-0.56	<0,05	-0.32	<0.05
GgaAffx.22958.1.S1_at	C2orf37	ENSGALG00000009558	7	19463512	19490565	0.66	<0,05	0.82	<0.001
GgaAffx.22467.1.S1_at	C2orf60	ENSGALG00000008145	7	12081567	12082666	-0.76	<0,05	-0.53	<0.01
GgaAffx.23939.1.S1_s_at	GPD2	ENSGALG00000012543	7	37689383	37741052	0.59	<0,05	0.52	<0.01
Gga.6263.2.S1_a_at	LOC424014	ENSGALG00000003797	7	4540068	4542656	0.58	<0,05	0.45	<0.05
Gga.9339.1.S1_at	MKI67IP	ENSGALG00000011650	7	27821268	27825051	-0.43	<0,05	-0.47	<0.01
GgaAffx.7286.1.S1_s_at	PECR	ENSGALG00000011497	7	25110149	25122516	-0.48	<0,05	-0.36	<0.05
Gga.12599.1.S1_s_at	WDR12	ENSGALG00000008470	7	13036443	13043789	-0.45	<0,05	-0.36	<0.05
GgaAffx.26704.1.S1_s_at	ABHD7	ENSGALG00000006019	8	14973233	14988992	-0.58	<0,01	-0.48	<0.01
Gga.8001.3.S1_at	ATPAF1	ENSGALG00000010444	8	22570693	22574048	-2.14	<0,001	-2.14	<0.001
Gga.16715.1.S1_at	EVI5	ENSGALG00000005935	8	14768562	14778550	-0.77	<0,01	-1.08	<0.001
Gga.1106.1.S1_at	FAM129A	ENSGALG00000004812	8	8276566	8277792	1.07	<0,05	0.51	<0.001
Gga.11829.1.S1_s_at	FGGY	ENSGALG00000010879	8	27331465	27400581	-0.66	<0,05	-0.65	<0.05
Gga.9018.3.S1_a_at	KIAA0494	ENSGALG00000010446	8	22575409	22576428	-0.40	<0,05	-0.41	<0.05
Gga.4092.1.S1_at	RCJMB04_1d5	ENSGALG00000010543	8	24800016	24808256	0.65	<0,05	0.67	<0.001
Gga.9632.1.S1_at	RPAP2	ENSGALG00000005953	8	14865147	14899775	-1.83	<0,01	-1.69	<0.001
GgaAffx.20452.1.S1_at	SNORD38	ENSGALG00000024758	8	21483520	21484351	-0.53	<0,05	-0.47	<0.05
Gga.11233.1.S1_at	WDR65	ENSGALG00000009902	8	20340795	20345708	0.57	<0,05	0.50	<0.01

GgaAffx.26046.1.S1_at	XPR1	ENSGALG00000003893	8	6142428	6160559	-0.38	<0,05	-0.33	<0.05
Gga.3340.1.S1_at	-	-	8	14571287	14572781	1.27	<0,001	1.26	<0.0001
GgaAffx.21718.1.S1_at	-	-	8	29145565	29158473	-0.40	<0,05	-0.58	<0.05
Gga.645.1.S1_s_at	SKIL	ENSGALG00000009356	9	21337078	21349669	0.98	<0,01	0.86	<0.01
Gga.14467.1.S1_at	-	-	9	23774155	23775162	-0.46	<0,05	-0.43	<0.05
GgaAffx.4000.2.S1_at	ARNT2	ENSGALG00000006445	10	14113250	14198775	-1.33	<0,01	-1.48	<0.001
Gga.7125.1.S1_a_at	DIS3L	ENSGALG00000007643	10	20619465	20620655	0.61	<0,05	0.75	<0.001
Gga.16228.1.S1_at	FAM81A	ENSGALG00000004103	10	7688166	7692981	-0.50	<0,05	-0.46	<0.01
Gga.12361.1.S1_at	RLBP1	ENSGALG00000006676	10	14662031	14666268	2.03	<0,01	1.65	<0.001
GgaAffx.13081.1.S1_s_at	SNAPC5	ENSGALG00000007695	10	20664687	20667113	-0.55	<0,01	-0.52	<0.001
GgaAffx.9087.2.S1_s_at	ARL2BP	ENSGALG00000014326	11	746011	748305	2.36	<0,05	2.30	<0.01
GgaAffx.2225.1.S1_s_at	AYTL1	ENSGALG00000003574	11	3630267	3657319	-0.51	<0,05	-0.76	<0.001
Gga.12161.1.S1_at	BEAN	ENSGALG00000005266	11	12353007	12394775	-0.79	<0,05	-0.92	<0.001
Gga.9829.1.S1_at	CA5B	ENSGALG00000005855	11	19967605	19980195	-3.41	<0,01	-3.89	<0.0001
Gga.7601.1.S1_at	GALNS	ENSGALG00000006282	11	20442252	20455777	-0.48	<0,05	-0.43	<0.01
Gga.8312.1.S1_at	LOC769704	ENSGALG00000021087	11	12000979	12014992	0.74	<0,01	0.78	<0.01
GgaAffx.12446.1.S1_at	TMEM208	ENSGALG00000001722	11	981693	994129	1.05	<0,001	0.73	<0.01
Gga.2607.3.S1_at	-	ENSGALG00000021463	11	901267	902611	1.02	<0,01	1.06	<0.001
Gga.6641.1.A1_at	-	-	11	1003472	1003877	0.51	<0,05	0.32	<0.05
Gga.9143.1.S1_x_at	-	-	11	20805306	20805967	-0.56	<0,01	-0.36	<0.01
GgaAffx.3442.2.S1_s_at	ARHGEF3	ENSGALG00000005481	12	8667295	8697223	-0.73	<0,05	-0.79	<0.05
Gga.3220.1.S1_at	HEMK1	ENSGALG00000002253	12	1648313	1677585	0.57	<0,05	0.62	<0.01
Gga.8022.1.S1_at	TMF1	ENSGALG00000013408	12	15707568	15709293	-0.68	<0,01	-0.62	<0.001
Gga.10986.1.S1_at	-	-	12	733978	737367	0.68	<0,05	0.77	<0.01
Gga.19124.1.S1_at	-	ENSGALG00000006280	12	10969124	10970972	1.18	<0,01	1.20	<0.001
GgaAffx.21429.1.S1_at	-	-	12	14031869	14032997	0.54	<0,05	0.47	<0.01
GgaAffx.12925.1.S1_at	SLU7	ENSGALG00000001500	13	7581375	7592262	0.46	<0,05	0.60	<0.001

Gga.17661.1.S1_s_at	-	ENSGALG00000004184	13	12997370	13001470	0.53	<0,05	0.59	<0.01
Gga.4567.3.S1_a_at	ATP5J2	ENSGALG00000004717	14	4408185	4409231	1.56	<0,001	1.48	<0.001
Gga.3226.1.S1_at	CRAMP1L	ENSGALG00000009341	14	14443149	14447337	0.61	<0,01	0.68	<0.05
Gga.9600.2.S1_at	DCTN5	ENSGALG00000006088	14	6928952	6936129	-0.70	<0,05	-0.59	<0.001
Gga.9001.1.S1_at	LOC426427	ENSGALG00000000687	14	12492211	12496904	-1.21	<0,05	-1.24	<0.0001
GgaAffx.12957.1.S1_at	NMRAL1	ENSGALG00000007827	14	13438988	13443917	0.72	<0,05	0.65	<0.001
Gga.8055.1.S1_a_at	-	ENSGALG00000024058	14	6019598	6020123	0.40	<0,05	0.28	<0.05
GgaAffx.13043.1.S1_s_at	C22orf25	ENSGALG00000002056	15	1276082	1288928	-0.99	<0,01	-0.83	<0.0001
Gga.5155.2.S1_a_at	GATC	ENSGALG00000007195	15	9554507	9555792	-0.36	<0,05	-0.41	<0.01
Gga.8061.1.S1_at	HSCB	ENSGALG00000005706	15	7935489	7939021	3.08	<0,05	2.44	<0.01
Gga.9711.2.S1_s_at	LOC416827	ENSGALG00000003413	15	5104671	5107136	-0.51	<0,05	-0.54	<0.01
Gga.2027.1.A1_at	POP5	ENSGALG00000007124	15	9516532	9517593	0.50	<0,05	0.46	<0.01
Gga.9053.1.S1_s_at	XBP1	ENSGALG00000005796	15	7957482	7960608	0.32	<0,05	0.42	<0.01
GgaAffx.5614.1.S1_at	NDOR1	ENSGALG00000008920	17	1342842	1359784	-0.41	<0,05	-0.31	<0.05
GgaAffx.11982.1.S1_at	CHMP6	ENSGALG00000006920	18	9334367	9340258	-0.66	<0,05	-0.50	<0.01
Gga.449.1.S1_at	NOG	ENSGALG00000003114	18	6194400	6195400	-0.53	<0,05	-0.49	<0.01
GgaAffx.25881.1.S1_s_at	PECAM1	ENSGALG00000003515	18	6870154	6878882	-0.84	<0,01	-0.73	<0.05
Gga.14171.1.S1_at	-	-	18	10297821	10299348	0.65	<0,01	0.62	<0.001
Gga.13199.1.S1_at	TAOK1	ENSGALG00000004082	19	5996010	6002563	-0.70	<0,05	-1.04	<0.001
Gga.14944.1.A1_at	-	-	19	8742382	8743474	0.52	<0,05	0.40	<0.01
Gga.17597.1.S1_at	-	-	19	8735422	8737394	-0.59	<0,05	-0.88	<0.001
Gga.17145.1.S1_at	C20orf142	ENSGALG00000004301	20	5435342	5440460	0.60	<0,05	0.50	<0.01
Gga.11650.2.S1_at	GCNT7	ENSGALG00000007717	20	11942120	11946284	-0.70	<0,05	-0.52	<0.05
Gga.5213.1.S1_a_at	LOC419144	ENSGALG00000001919	20	1418494	1420388	-0.41	<0,05	-0.45	<0.05
Gga.11229.1.S1_at	PPP1R3D	ENSGALG00000021220	20	6840004	6841126	1.24	<0,01	1.24	<0.001
Gga.17872.1.S1_at	-	-	20	5869614	5870277	-1.19	<0,05	-1.03	<0.001
Gga.11835.1.S1_at	C1orf187	ENSGALG00000004631	21	5834624	5841301	-0.40	<0,05	-0.37	<0.01

Gga.1067.1.S1_a_at	-	ENSGALG00000024115	21	5944459	5946037	0.74	<0,05	0.80	<0.001
Gga.16295.1.S1_at	-	-	21	3475643	3476782	-1.06	<0,05	-1.32	<0.0001
Gga.6140.1.A1_s_at	PCYOX1	ENSGALG00000013878	22	2890411	2893520	0.44	<0,05	0.22	<0.05
Gga.6045.1.S1_at	-	-	22	3130121	3326494	0.58	<0,05	0.52	<0.01
GgaAffx.8817.1.A1_at	-	-	22	2852406	2853087	-0.52	<0,05	-0.74	<0.001
Gga.2537.2.S1_at	HMGCL	ENSGALG00000004057	23	5869872	5871780	0.61	<0,05	0.49	<0.01
Gga.11888.1.S1_at	CCDC84	ENSGALG00000007689	24	5778171	5781505	-0.95	<0,05	-1.01	<0.01
Gga.7980.1.S1_at	IRF6	ENSGALG00000001405	26	2989457	2993869	-0.55	<0,05	-0.67	<0.001
Gga.15115.1.S1_at	CERK	ENSGALG00000019397	Un_random	15817526	15818086	-0.36	<0,05	-0.38	<0.01
GgaAffx.20317.1.S1_at	LOC425455	ENSGALG00000018770	Un_random	28281655	28282534	-0.49	<0,05	-0.38	<0.01
Gga.16292.1.S1_at	TGOLN2	ENSGALG00000014429	Un_random	60211708	60215651	0.59	<0,05	0.61	<0.01
Gga.16613.1.S1_at	-	-	Un_random	54903400	54904287	0.86	<0,01	1.06	<0.01
Gga.20015.1.S1_at	-	-	Un_random	10970350	10970790	-1.43	<0,001	-1.50	<0.0001
Gga.4900.5.S1_at	-	-	Un_random	36367609	36370737	1.07	<0,05	0.86	<0.001
Gga.8013.1.S1_at	-	-	Un_random	5373553	5377650	-0.65	<0,05	-0.49	<0.05
GgaAffx.21198.1.S1_at	-	-	Un_random	29867459	29869475	0.61	<0,05	0.50	<0.01
GgaAffx.6391.2.S1_s_at	ANKRD15	ENSGALG00000010158	Z	26696084	26721790	-0.52	<0,05	-0.63	<0.001
Gga.318.1.S1_at	KCNN2	ENSGALG00000002539	Z	71359953	71432785	-0.39	<0,05	-0.35	<0.05
Gga.2531.1.S1_at	LHFPL2	ENSGALG00000004437	Z	22148376	22152205	-1.02	<0,01	-0.94	<0.05
Gga.8239.1.S1_at	LOC427229	-	Z	28415501	28420860	0.57	<0,05	0.63	<0.01
Gga.7044.3.S1_s_at	SEPP1	ENSGALG00000014857	Z	13011276	13013632	0.52	<0,05	0.68	<0.01
Gga.7337.1.S1_at	SSBP2	ENSGALG00000023089	Z	62191512	62361161	-1.03	<0,01	-0.99	<0.01
Gga.59.1.S1_at	LOC395260	-				0.46	<0,05	0.63	<0.01
Gga.4308.1.S1_at	LOC396507	-				1.76	<0,01	1.84	<0.0001
Gga.19447.2.S1_s_at	LOC770705	-				1.97	<0,05	2.33	<0.01
Gga.11404.1.S1_at	-	-				2.08	<0,05	1.98	<0.01
Gga.15366.1.S1_at	-	-				-2.35	<0,01	-2.32	<0.01

Gga.15888.1.S1_s_at	-	-	-0.43	<0,05	-0.72	<0.001
Gga.19925.1.S1_at	-	-	1.37	<0,05	1.21	<0.01
Gga.2315.1.S1_s_at	-	-	-0.77	<0,05	-0.75	<0.0001
Gga.7474.1.S1_at	-	-	-0.50	<0,05	-0.54	<0.01
Gga.8848.1.S1_at	-	-	1.21	<0,01	0.98	<0.001
GgaAffx.12910.1.S1_at	-	-	0.94	<0,05	1.16	<0.01

* Differential gene expression is given as: FC = fold change logarithm to the base of 2 (log2), adj.P = Bayes moderated t-test false discovery rate adjusted p-value.
