# Supplementary material for: Heritable genome-wide variation of gene expression and promoter methylation between wild and domesticated chickens
Source: BMC Genomics. 2012 Feb 4;13:59. doi: 10.1186/1471-2164-13-59 (PMC3297523; doi:10.1186/1471-2164-13-59)
Supplement: Additional file 4 — Expression and methylation. Gene expression differences plotted against promoter methylation differences between WL and RJF offspring. [file 1471-2164-13-59-S4.PDF]

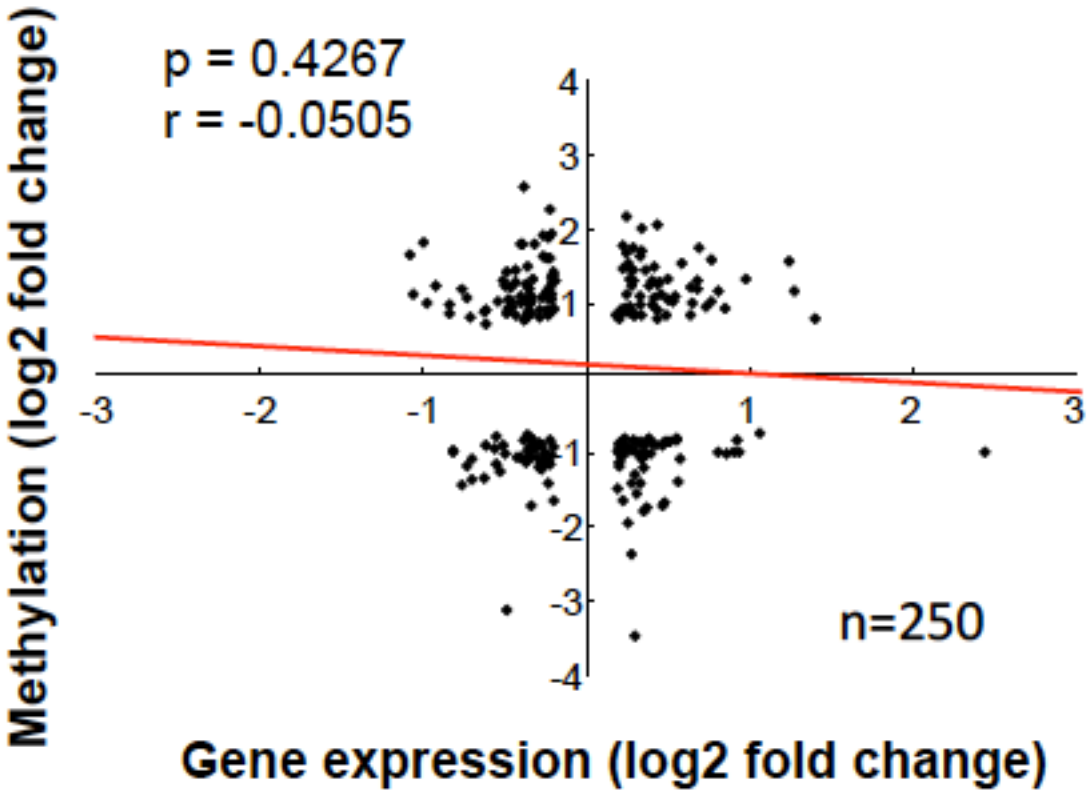

Additional file 4. Gene expression difference between WL and RJF offspring plotted against difference in methylation of the promoters of the same genes. The graph includes the 250 genes which were both significantly differentially expressed and methylated (FDR-corrected P<0.05)
