# Supplementary material for: Heritable genome-wide variation of gene expression and promoter methylation between wild and domesticated chickens
Source: BMC Genomics. 2012 Feb 4;13:59. doi: 10.1186/1471-2164-13-59 (PMC3297523; doi:10.1186/1471-2164-13-59)
Supplement: Additional file 5 — Differentially methylated genes. A full list of all genes, where the promotors were found to be differentially methylated, in both generations. [file 1471-2164-13-59-S5.PDF]

**Additional file 5 | Differentially methylated promotors of individual genes, comparing breeds, in both generations. The table gives the Ensembl ID and the name of the gene, where available. For each gene the probe genomic coordinates are shown, and the fold change (FC; negative sign shows lower value in domestic birds) together with the FDR corrected P-value (adj.P). For each probe, its GC-content and nrs of SNPs are given.**

| *Identifiers* | | *Probe genomic coordinates* | | | *Probe features* | | *Parents** | | *Offspring** | |
|---|---|---|---|---|---|---|---|---|---|---|
| **Gene (s)** | **Ensembl Gene ID (s)** | **Chromosome** | **Start** | **End** | **GC%** | **SNP** | **FC** | **adj. P** | **FC** | **adj.P** |
| FAM19A5 | ENSGALG00000023584 | 1 | 18457335 | 18457397 | 0.37 | No | 1.29 | <0.05 | 1.09 | <0.05 |
| BRD1 | ENSGALG00000008526 | 1 | 20062710 | 20062768 | 0.34 | No | -1.09 | <0.05 | -1.43 | <0.01 |
| LOC429348 | ENSGALG00000005013 | 1 | 21595421 | 21595471 | 0.72 | No | 1.73 | <0.05 | 1.77 | <0.01 |
| MAPK11 | ENSGALG00000008612 | 1 | 21835058 | 21835108 | 0.48 | Yes (2) | 1.25 | <0.05 | 1.12 | <0.01 |
| TMEM184B | ENSGALG00000012272 | 1 | 52894935 | 52894985 | 0.72 | No | 2.01 | <0.01 | 0.97 | <0.05 |
| HMOX1 | ENSGALG00000012550 | 1 | 54145310 | 54145360 | 0.6 | No | 1.54 | <0.05 | 1.48 | <0.01 |
| KRAS | ENSGALG00000014033 | 1 | 69417124 | 69417174 | 0.72 | No | 1.93 | <0.05 | 1.64 | <0.001 |
| STK38L | ENSGALG00000014094 | 1 | 70239290 | 70239340 | 0.52 | No | 2.4 | <0.01 | 2.16 | <0.01 |
| (No name);LOC768852 | ENSGALG00000021752; ENSGALG00000021740 | 1 | 104459213 | 104459263 | 0.6 | No | -2.09 | <0.05 | -1.67 | <0.05 |
| (No name) | ENSGALG00000022847 | 1 | 104491892 | 104491942 | 0.78 | No | 1.67 | <0.05 | 2.04 | <0.0001 |
| CCT8 | ENSGALG00000015821 | 1 | 107474471 | 107474521 | 0.58 | Yes (1) | 2.1 | <0.05 | 1.28 | <0.05 |
| NR0B1 | ENSGALG00000016287 | 1 | 119394904 | 119394954 | 0.54 | No | 2.58 | <0.05 | 2.09 | <0.05 |
| CNKSR2 | ENSGALG00000016391 | 1 | 122809631 | 122809685 | 0.39 | No | -1.54 | <0.05 | -1.26 | <0.05 |
| PPP2R3B | ENSGALG00000016702 | 1 | 134058725 | 134058781 | 0.39 | No | 1.25 | <0.05 | 0.92 | <0.05 |
| GAS6 | ENSGALG00000016820 | 1 | 141412612 | 141412662 | 0.46 | No | 1.21 | <0.05 | 0.85 | <0.05 |
| C13orf7 | ENSGALG00000016911 | 1 | 157578139 | 157578189 | 0.46 | Yes (1) | 1.89 | <0.01 | 1.14 | <0.05 |
| U6 | ENSGALG00000017900 | 1 | 188045916 | 188045974 | 0.4 | No | -1.34 | <0.05 | -1.80 | <0.001 |
| MRE11A | ENSGALG00000017211 | 1 | 189909613 | 189909672 | 0.39 | No | 1.82 | <0.05 | 1.32 | <0.05 |
| RRM1 | ENSGALG00000017350 | 1 | 199563532 | 199563582 | 0.54 | No | 1.89 | <0.01 | 1.33 | <0.01 |
| MRPL48 | ENSGALG00000017319 | 1 | 200511122 | 200511172 | 0.52 | No | 2.01 | <0.01 | 2.19 | <0.001 |
| ICER | ENSGALG00000007082 | 2 | 12936045 | 12936102 | 0.4 | No | 1.12 | <0.05 | 0.81 | <0.05 |
| RUNDC3B | ENSGALG00000008954 | 2 | 20709363 | 20709413 | 0.5 | No | 1.83 | <0.01 | 1.37 | <0.05 |
| TMEM106B | ENSGALG00000010720 | 2 | 26550633 | 26550693 | 0.33 | Yes (1) | 1.44 | <0.05 | 1.39 | <0.05 |
| ELMO1 | ENSGALG00000012093 | 2 | 46394319 | 46394369 | 0.54 | No | 1.06 | <0.05 | 1.01 | <0.05 |
| ELOVL2 | ENSGALG00000012748 | 2 | 64288196 | 64288249 | 0.42 | Yes (1) | 2.02 | <0.01 | 1.05 | <0.05 |
| PECI | ENSGALG00000012809 | 2 | 67086639 | 67086689 | 0.54 | Yes (1) | 1.7 | <0.05 | 1.14 | <0.05 |
| C6orf85 | ENSGALG00000012816 | 2 | 67366315 | 67366365 | 0.66 | No | 1.52 | <0.05 | 1.26 | <0.01 |

| | | | | | | | | | | |
|---|---|---|---|---|---|---|---|---|---|---|
| BPHL | ENSGALG00000012824 | 2 | 67519887 | 67519939 | 0.42 | No | 1.73 | <0.05 | 1.55 | <0.001 |
| NQO2 | ENSGALG00000012828 | 2 | 67575666 | 67575716 | 0.48 | Yes (3) | 1.83 | <0.01 | 1.57 | <0.001 |
| SERPINB14B | ENSGALG00000012871 | 2 | 68934717 | 68934767 | 0.54 | No | -1.03 | <0.05 | -0.89 | <0.05 |
| C6orf62 | ENSGALG00000013628 | 2 | 92562269 | 92562319 | 0.6 | Yes (1) | 1.34 | <0.05 | 1.76 | <0.01 |
| LYN | ENSGALG00000018967 | 2 | 114788007 | 114788057 | 0.52 | Yes (1) | -1.65 | <0.01 | -1.50 | <0.01 |
| (No name);FAM82B | ENSGALG00000018073; ENSGALG00000022790 | 2 | 127865016 | 127865066 | 0.5 | No | 1.53 | <0.05 | 1.15 | <0.05 |
| RPLP1 | ENSGALG00000016172 | 2 | 151341582 | 151341632 | 0.66 | Yes (1) | 1.89 | <0.01 | 2.19 | <0.0001 |
| GNMT | ENSGALG00000008575 | 3 | 16946697 | 16946751 | 0.43 | Yes (4) | -1.62 | <0.05 | -1.64 | <0.05 |
| GALM | ENSGALG00000013828 | 3 | 17506672 | 17506722 | 0.5 | Yes (1) | -1.95 | <0.01 | -1.59 | <0.01 |
| FLVCR1 | ENSGALG00000009807 | 3 | 22929278 | 22929328 | 0.54 | Yes (1) | -2.17 | <0.01 | -2.48 | <0.0001 |
| KCNK1 | ENSGALG00000011005 | 3 | 40424820 | 40424870 | 0.72 | No | 1.73 | <0.05 | 1.83 | <0.01 |
| LOC769676 | ENSGALG00000013752 | 3 | 54160293 | 54160343 | 0.82 | No | 1.51 | <0.01 | 1.37 | <0.01 |
| PHACTR2 | ENSGALG00000013768 | 3 | 54276682 | 54276735 | 0.32 | No | -1.22 | <0.05 | -1.62 | <0.01 |
| C6orf170 | ENSGALG00000014876 | 3 | 64463401 | 64463451 | 0.74 | No | 1.62 | <0.05 | 1.69 | <0.01 |
| COL12A1 | ENSGALG00000015908 | 3 | 83548442 | 83548492 | 0.84 | No | 1.43 | <0.05 | 1.10 | <0.05 |
| EIF2B4; LOC421988 | ENSGALG00000012471; ENSGALG00000016514 | 3 | 107369434 | 107369484 | 0.56 | Yes (1) | -1.62 | <0.05 | -1.94 | <0.001 |
| C2orf18 | ENSGALG00000016589 | 3 | 108007337 | 108007387 | 0.54 | No | 1.94 | <0.01 | 1.69 | <0.001 |
| CXorf9 | ENSGALG00000003951 | 4 | 1626506 | 1626556 | 0.7 | No | 1.92 | <0.05 | 1.83 | <0.01 |
| GLA; BTK; GLRA4 | ENSGALG00000004948; ENSGALG00000004958; ENSGALG00000004936 | 4 | 1997394 | 1997444 | 0.78 | No | 1.4 | <0.05 | 1.08 | <0.05 |
| SNORD61; RBMX | ENSGALG00000025014; ENSGALG00000006457 | 4 | 4430506 | 4430556 | 0.48 | No | 1.19 | <0.05 | 1.03 | <0.05 |
| ATP11C | ENSGALG00000006623 | 4 | 5135711 | 5135761 | 0.52 | Yes (2) | 1.36 | <0.05 | 1.31 | <0.01 |
| (DDX60) | ENSGALG00000009639 | 4 | 26083200 | 26083265 | 0.23 | No | 1.95 | <0.05 | 1.97 | <0.0001 |
| USP38 | ENSGALG00000009886 | 4 | 31557525 | 31557576 | 0.43 | Yes (2) | -1.04 | <0.05 | -1.20 | <0.01 |
| GAB1 | ENSGALG00000009898 | 4 | 31682171 | 31682225 | 0.41 | Yes (2) | -1.49 | <0.01 | -1.72 | <0.001 |
| PIGY | ENSGALG00000023319 | 4 | 36198258 | 36198308 | 0.44 | No | -2.69 | <0.01 | -2.61 | <0.0001 |
| SLC39A8 | ENSGALG00000012298 | 4 | 62566143 | 62566193 | 0.48 | No | 2.12 | <0.001 | 1.44 | <0.001 |
| SOD3 | ENSGALG00000018557 | 4 | 76182138 | 76182188 | 0.54 | No | -1.96 | <0.01 | -1.56 | <0.01 |
| ADRA2C | ENSGALG00000015608 | 4 | 84593087 | 84593137 | 0.64 | No | 1.42 | <0.05 | 1.23 | <0.01 |
| DGKZ | ENSGALG00000008380 | 5 | 25845602 | 25845652 | 0.66 | No | 1.7 | <0.05 | 1.28 | <0.05 |
| VPS39 | ENSGALG00000008991 | 5 | 27777500 | 27777550 | 0.54 | Yes (4) | 1.88 | <0.05 | 1.60 | <0.01 |
| NUMB | ENSGALG00000009300 | 5 | 28372151 | 28372201 | 0.54 | No | 2.19 | <0.01 | 1.72 | <0.001 |
| NPC2 | ENSGALG00000010237 | 5 | 40333073 | 40333123 | 0.54 | No | 2.28 | <0.01 | 1.79 | <0.01 |
| MLH3; ACYP1 | ENSGALG00000010304; ENSGALG00000010307 | 5 | 40561605 | 40561655 | 0.52 | Yes (1) | 1.26 | <0.05 | 1.18 | <0.01 |
| LGMN | ENSGALG00000010811 | 5 | 47287023 | 47287074 | 0.43 | Yes (1) | 1.61 | <0.01 | 1.11 | <0.05 |

| | | | | | | | | | | |
|---|---|---|---|---|---|---|---|---|---|---|
| WDR20 | ENSGALG00000011365 | 5 | 52003300 | 52003360 | 0.35 | Yes (1) | -2.36 | <0.05 | -2.52 | <0.0001 |
| TXNDC16; GPR137C | ENSGALG00000012400; ENSGALG00000012401 | 5 | 60762358 | 60762408 | 0.54 | No | 1.44 | <0.05 | 1.25 | <0.05 |
| OIT3 | ENSGALG00000004345 | 6 | 12342000 | 12342050 | 0.48 | No | 1.99 | <0.01 | 2.12 | <0.001 |
| ZFYVE27; C10orf83 | ENSGALG00000007531; ENSGALG00000006149 | 6 | 23738612 | 23738662 | 0.58 | No | 1.16 | <0.05 | 1.66 | <0.001 |
| SFXN4 | ENSGALG00000009350 | 6 | 31510021 | 31510071 | 0.74 | No | 1.55 | <0.01 | 1.40 | <0.01 |
| SLC11A1; VIL1;TNS1 | ENSGALG00000011434; ENSGALG00000011433; ENSGALG00000011462 | 7 | 24097265 | 24097315 | 0.62 | No | 1.26 | <0.05 | 1.26 | <0.01 |
| ZRANB3; R3HDM1 | ENSGALG00000012223; ENSGALG00000012246 | 7 | 32167420 | 32167470 | 0.56 | No | 1.41 | <0.05 | 1.11 | <0.01 |
| LHX9 | ENSGALG00000002223 | 8 | 2303719 | 2303769 | 0.76 | No | 1.83 | <0.05 | 1.52 | <0.01 |
| LOC424386 | ENSGALG00000003081 | 8 | 4572647 | 4572697 | 0.7 | No | 1.42 | <0.05 | 1.29 | <0.01 |
| ABHD7 | ENSGALG00000006019 | 8 | 14986410 | 14986460 | 0.46 | No | -3.06 | <0.001 | -2.98 | <0.0001 |
| DDAH1 | ENSGALG00000008663 | 8 | 16980025 | 16980080 | 0.33 | Yes (1) | 1.73 | <0.01 | 2.54 | <0.0001 |
| TMEM125 | ENSGALG00000009951 | 8 | 20350483 | 20350533 | 0.74 | No | 2.01 | <0.01 | 1.67 | <0.01 |
| KLF1 | ENSGALG00000010101 | 8 | 21120173 | 21120223 | 0.52 | Yes (3) | 2.2 | <0.01 | 1.53 | <0.001 |
| C1orf163 | ENSGALG00000010637 | 8 | 25254877 | 25254927 | 0.66 | Yes (1) | 2.43 | <0.05 | 1.93 | <0.001 |
| RAVER2 | ENSGALG00000011026 | 8 | 28963440 | 28963490 | 0.54 | No | 1.61 | <0.01 | 1.52 | <0.001 |
| FOXL2 | ENSGALG00000021009 | 9 | 6763953 | 6764003 | 0.84 | No | 1.97 | <0.001 | 1.63 | <0.001 |
| IGSF10 | ENSGALG00000010369 | 9 | 25053375 | 25053425 | 0.54 | No | 1.41 | <0.05 | 1.09 | <0.01 |
| LRRC28 | ENSGALG00000007057 | 10 | 18946642 | 18946692 | 0.68 | Yes (1) | 1.82 | <0.01 | 1.31 | <0.01 |
| C16orf70 | ENSGALG00000003183 | 11 | 2379353 | 2379405 | 0.35 | No | -1.86 | <0.05 | -1.18 | <0.01 |
| FBXL8 | ENSGALG00000003201 | 11 | 2444150 | 2444200 | 0.68 | No | 1.59 | <0.05 | 1.31 | <0.01 |
| PEPD | ENSGALG00000004899 | 11 | 11175122 | 11175173 | 0.43 | No | -1.09 | <0.05 | -1.21 | <0.01 |
| BEAN | ENSGALG00000005266 | 11 | 12395420 | 12395470 | 0.7 | No | 1.92 | <0.05 | 1.25 | <0.05 |
| WWP2; NQO1; TERF2 | ENSGALG00000000699; ENSGALG00000023437; ENSGALG00000000657 | 11 | 21003698 | 21003748 | 0.6 | No | 1.93 | <0.05 | 1.82 | <0.01 |
| SLMAP | ENSGALG00000005670 | 12 | 8993934 | 8993994 | 0.38 | Yes (2) | -1.47 | <0.05 | -1.41 | <0.01 |
| CCDC72 | ENSGALG00000006463 | 12 | 11565598 | 11565648 | 0.76 | No | 2.02 | <0.01 | 1.79 | <0.01 |
| EIF4E3 | ENSGALG00000007780 | 12 | 16883629 | 16883679 | 0.66 | No | 1.66 | <0.05 | 1.76 | <0.01 |
| IFT122 | ENSGALG00000008452 | 12 | 20123112 | 20123162 | 0.72 | No | 1.73 | <0.05 | 1.50 | <0.01 |
| PCDHAC1,2 | ENSGALG00000000760 | 13 | 699620 | 699677 | 0.4 | Yes (4) | 1.3 | <0.05 | 1.21 | <0.01 |
| PPP2CA | ENSGALG00000006462 | 13 | 16333089 | 16333146 | 0.4 | No | 2.19 | <0.05 | 2.19 | <0.01 |
| LOC776047 | ENSGALG00000006569 | 13 | 17043933 | 17043985 | 0.37 | No | 1.63 | <0.05 | 1.26 | <0.01 |
| GFRA3 | ENSGALG00000007620 | 13 | 18727018 | 18727078 | 0.4 | No | -1.63 | <0.05 | -1.88 | <0.0001 |
| IQCE | ENSGALG00000004329 | 14 | 3348891 | 3348948 | 0.4 | No | 2.23 | <0.01 | 1.58 | <0.01 |
| LOC425593 | ENSGALG00000010497 | 14 | 4106867 | 4106917 | 0.64 | Yes (1) | 1.35 | <0.05 | 1.18 | <0.05 |
| ATP5J2; PTCD1 | ENSGALG00000004717; ENSGALG00000004711 | 14 | 4405782 | 4405832 | 0.46 | No | 1.59 | <0.01 | 1.42 | <0.001 |

| | | | | | | | | | | |
|---|---|---|---|---|---|---|---|---|---|---|
| B9D1 | ENSGALG00000005096 | 14 | 5251957 | 5252007 | 0.62 | No | 1.74 | <0.05 | 1.60 | <0.05 |
| ABCA3 | ENSGALG00000001967 | 14 | 14359837 | 14359887 | 0.62 | Yes (2) | 1.3 | <0.05 | 1.01 | <0.05 |
| VRK3 | ENSGALG00000020725 | 14 | 15044969 | 15045019 | 0.72 | No | -2.69 | <0.001 | -2.16 | <0.001 |
| CRYBB3 | ENSGALG00000005515 | 15 | 7212832 | 7212882 | 0.6 | No | -1.75 | <0.05 | -1.52 | <0.05 |
| C22orf13 | ENSGALG00000006607 | 15 | 8680903 | 8680968 | 0.35 | No | 1.69 | <0.01 | 2.18 | <0.001 |
| TBC1D10A | ENSGALG00000007954 | 15 | 11177311 | 11177361 | 0.66 | Yes (1) | 1.26 | <0.05 | 0.99 | <0.05 |
| TESC | ENSGALG00000008206 | 15 | 11654121 | 11654171 | 0.58 | No | 2 | <0.01 | 1.31 | <0.01 |
| GNB2L1; TRIM41; SNORD95 | ENSGALG00000000122; ENSGALG00000019835; ENSGALG00000017829 | 16 | 104002 | 104052 | 0.62 | No | 2.3 | <0.05 | 1.69 | <0.01 |
| RAPGEF1 | ENSGALG00000003712 | 17 | 6953715 | 6953765 | 0.7 | No | 1.83 | <0.05 | 1.27 | <0.05 |
| STX8 | ENSGALG00000001238 | 18 | 2124355 | 2124405 | 0.52 | No | 1.17 | <0.05 | 0.76 | <0.05 |
| HLF | ENSGALG00000003059 | 18 | 5907124 | 5907181 | 0.4 | No | 2.21 | <0.01 | 1.90 | <0.001 |
| UTP6 | ENSGALG00000003260 | 18 | 6585238 | 6585288 | 0.8 | No | 1.91 | <0.01 | 1.45 | <0.01 |
| PDE6G; NPLOC4 | ENSGALG00000004514; ENSGALG00000004583 | 18 | 9173269 | 9173319 | 0.62 | No | 1.2 | <0.05 | 0.91 | <0.05 |
| C17orf85 | ENSGALG00000002653 | 19 | 5129436 | 5129486 | 0.44 | Yes (1) | 1.55 | <0.05 | 1.25 | <0.05 |
| SERPINF2 | ENSGALG00000002987 | 19 | 5363530 | 5363580 | 0.46 | No | -1.97 | <0.05 | -1.80 | <0.001 |
| MYO19 | ENSGALG00000005374 | 19 | 8096951 | 8097001 | 0.74 | No | 1.63 | <0.05 | 1.07 | <0.05 |
| LHX1 | ENSGALG00000005409 | 19 | 8343558 | 8343608 | 0.54 | No | 1.97 | <0.001 | 1.52 | <0.01 |
| KSR1 | ENSGALG00000005685 | 19 | 9096187 | 9096237 | 0.48 | No | -1.46 | <0.05 | -1.27 | <0.01 |
| gga-mir-1689; MNT | ENSGALG00000025334; ENSGALG00000005830 | 19 | 9371568 | 9371618 | 0.76 | No | 1.4 | <0.05 | 1.39 | <0.01 |
| RAP1GAP2 | ENSGALG00000005868 | 19 | 9503195 | 9503245 | 0.62 | No | 1.56 | <0.01 | 1.48 | <0.01 |
| MATN4 | ENSGALG00000004028 | 20 | 5125683 | 5125733 | 0.64 | No | 1.48 | <0.01 | 1.16 | <0.01 |
| NCOA3 | ENSGALG00000004570 | 20 | 5852915 | 5852972 | 0.4 | No | 1.63 | <0.05 | 1.45 | <0.05 |
| PLTP | ENSGALG00000006894 | 20 | 10496653 | 10496703 | 0.66 | Yes (2) | 1.77 | <0.05 | 1.34 | <0.05 |
| ELMO2 | ENSGALG00000007246 | 20 | 10701274 | 10701324 | 0.8 | No | 1.68 | <0.05 | 1.66 | <0.01 |
| STX16 | ENSGALG00000007541 | 20 | 11037743 | 11037793 | 0.56 | No | 1.92 | <0.05 | 1.38 | <0.05 |
| ZBTB48 | ENSGALG00000000637 | 21 | 556982 | 557032 | 0.76 | No | 1.84 | <0.01 | 1.75 | <0.01 |
| DRAXI | ENSGALG00000004631 | 21 | 5846446 | 5846496 | 0.68 | No | 1.45 | <0.05 | 1.27 | <0.01 |
| DNAJC8 | ENSGALG00000000790 | 23 | 1508266 | 1508316 | 0.48 | Yes (1) | 2.62 | <0.01 | 1.29 | <0.05 |
| FNDC5 | ENSGALG00000003567 | 23 | 5554910 | 5554960 | 0.82 | No | 1.54 | <0.05 | 1.45 | <0.01 |
| FUCA1; HMGCL | ENSGALG00000004112; ENSGALG00000004057 | 23 | 5876505 | 5876555 | 0.5 | No | 1.42 | <0.05 | 1.39 | <0.01 |
| IL18 | ENSGALG00000007874 | 24 | 6286869 | 6286919 | 0.5 | No | 1.64 | <0.01 | 1.80 | <0.001 |
| UBQLN4 | ENSGALG00000014022 | 25 | 159908 | 159958 | 0.46 | Yes (2) | 2.11 | <0.05 | 2.14 | <0.001 |
| CNTN2 | ENSGALG00000000653 | 26 | 1777093 | 1777143 | 0.52 | Yes (1) | -1.8 | <0.05 | -1.71 | <0.01 |
| CAMK1G | ENSGALG00000001319 | 26 | 2919443 | 2919493 | 0.78 | Yes (1) | 2.01 | <0.01 | 1.63 | <0.05 |

| | | | | | | | | | | |
|---|---|---|---|---|---|---|---|---|---|---|
| MOV10 | ENSGALG00000001558 | 26 | 3368527 | 3368584 | 0.4 | No | 2.55 | <0.01 | 2.43 | <0.01 |
| (No name);BTG2 | ENSGALG00000023753; ENSGALG00000003535 | 26 | 4939391 | 4939441 | 0.56 | No | 1.54 | <0.01 | 1.57 | <0.01 |
| (No name, Ig-like) | ENSGALG00000024250 | 27 | 229171 | 229221 | 0.76 | No | 2.53 | <0.05 | 1.98 | <0.05 |
| LMNB2; RPL36 | ENSGALG00000000470; ENSGALG00000000474 | 28 | 647994 | 648044 | 0.64 | No | 1.89 | <0.05 | 1.19 | <0.05 |
| GAMT; DAZAP1 | ENSGALG00000024304; ENSGALG00000015200 | 28 | 2525649 | 2525699 | 0.64 | Yes (2) | 1.67 | <0.01 | 1.34 | <0.01 |
| PTPRS | ENSGALG00000004048 | 28 | 4198021 | 4198071 | 0.6 | No | 1.76 | <0.05 | 1.40 | <0.05 |
| SAFB2 | ENSGALG00000013365; ENSGALG00000001948 | 28_random | 17449 | 17499 | 0.46 | - | -1.29 | <0.05 | -1.25 | <0.01 |
| (No name) | ENSGALG00000024020 | E64 | 21593 | 21654 | 0.38 | - | -2.81 | <0.001 | -2.23 | <0.01 |
| (No name, Ig-like) | ENSGALG00000000662; ENSGALG00000022500; ENSGALG00000022501 | Un_random | 15185723 | 15185773 | 0.52 | - | 2.9 | <0.001 | 1.94 | <0.001 |
| NRBP2 | ENSGALG00000000076 | Un_random | 55743548 | 55743598 | 0.48 | - | 1.18 | <0.05 | 1.19 | <0.05 |
| AP2S1 | ENSGALG00000000439 | Un_random | 63229163 | 63229213 | 0.46 | - | -1.91 | <0.05 | -1.42 | <0.01 |
| LOC427440 | ENSGALG00000003629 | Z | 10859990 | 10860051 | 0.36 | - | 2.88 | <0.001 | 2.24 | <0.001 |
| ERBB2IP | ENSGALG00000014773 | Z | 20142560 | 20142610 | 0.66 | - | 1.45 | <0.05 | 1.28 | <0.05 |
| CFC1B | ENSGALG00000012623 | Z | 41918552 | 41918610 | 0.4 | - | -1.44 | <0.05 | -1.70 | <0.01 |
| - | ENSGALG00000023087 | Z | 62579792 | 62579842 | 0.54 | - | 2.42 | <0.01 | 2.43 | <0.0001 |

SNP = Possible single nucleotide polymorphism detected in probe (numbers in brackets).
* Differential methylation is given as: FC = fold change logarithm to the base of 2 (log2), and adj.P = Bayes moderated t-test with false discovery rate adjusted p-value.
