# Supplementary material for: Heritable genome-wide variation of gene expression and promoter methylation between wild and domesticated chickens
Source: BMC Genomics. 2012 Feb 4;13:59. doi: 10.1186/1471-2164-13-59 (PMC3297523; doi:10.1186/1471-2164-13-59)
Supplement: Additional file 6 — Gene function. Gene ontology and KEGG pathway annotation for the genes which were either differentially expressed or differentially methylated in both generations, comparing between breeds. [file 1471-2164-13-59-S6.PDF]

Additional file 6| Gene ontology and KEGG pathway annotation for the genes which were either differentially expressed or differentially methylated in both generations, comparing between breeds.

Functional category	***Gene expression***		***DNA-methylation***	
	n	*p-value*	*n*	*p-value*
Parents and offspring	n_{tot}=149		n_{tot}=127	
Biological function				
Intracellular signaling cascade	17	0.06	15	<0.05
Ras protein signal transduction	4	0.06	5	<0.01
Parents	n_{tot}=159		n_{tot}=214	
Biological function				
Phosphorus metabolic process	14	0.07	19	<0.05
Phosphate metabolic process	14	0.07	19	<0.05
Intracellular signaling cascade	18	<0.05	23	<0.05
Protein kinase cascade	8	<0.05	9	0.07
Ras protein signal transduction	4	0.06	5	<0.05
KEGG-pathway				
MAPK signaling pathway	6	0.08	8	0.06
Offspring	n_{tot}=910		n_{tot}=705	
Biological function				
Protein amino acid phosphorylation	45	<0.05	40	<0.01
Coenzyme metabolic process	15	<0.05	11	0.07

Phosphorus metabolic process	62	<0.05	53	<0.01
Phosphate metabolic process	62	<0.05	53	<0.01
Lipid biosynthetic process	23	0.08	19	0.08
Response to metal ion	12	0.06	11	<0.05
Phosphorylation	50	0.08	40	<0.01
Positive regulation of kinase activity	21	<0.05	17	<0.05
Regulation of kinase activity	21	<0.05	17	<0.05
Myoblast differentiation	4	0.07	5	<0.01
Positive regulation of protein kinase activity	20	<0.05	16	<0.05
Cofactor metabolic process	16	0.06	13	0.07
Positive regulation of transferase activity	21	<0.05	17	<0.05
Oxidation reduction	43	<0.05	33	0.08
KEGG-pathways				
MAPK signaling pathway	22	0.09	18	0.06
Long-term potentiation	9	<0.05	9	<0.01
Neurotrophin signaling pathway	13	<0.05	11	<0.05
GnRH signaling pathway	11	<0.05	9	<0.05

n_{tot} = number of human homologs found in DAVID annotation database

n= number of genes in each functional category
