# Supplementary material for: Heritable genome-wide variation of gene expression and promoter methylation between wild and domesticated chickens
Source: BMC Genomics. 2012 Feb 4;13:59. doi: 10.1186/1471-2164-13-59 (PMC3297523; doi:10.1186/1471-2164-13-59)
Supplement: Additional file 7 — Selective sweep representation. Genes which were differentially expressed or methylated between breeds in both generations, and significantly overrepresented in selective sweeps associated with domestication. [file 1471-2164-13-59-S7.PDF]

Additional file 7 | Genes which were differentially expressed or methylated between breeds in both generations, and significantly overrepresented in selective sweeps.

Symbol	Gene name
Gene expression	
ABHD7	Abhydrolase domain containing 7
ANGEL1	Angel homolog 1
CPE	Carboxypeptidase E
GOPC	Golgi associated PDZ and coiled-coil motif
RPAP2	RNA polymerase II associated protein 2
TXNDC13	Thioredoxin domain containing 13
-	(GgaAffx.20610.1.S1_at)#
-	(Gga.3070.1.S1_at)#
-	(Gga.15291.1.S1_at)#
DNA-methylation	
ABHD7	Abhydrolase domain containing 7
ADRA2C	Adrenergic alpha-2C-receptor
FBXL8	F-box and leucine-rich repeat protein 8
KSR1	Kinase suppressor of ras 1
PTPRS	Protein tyrosine phosphatase receptor S
-	(ENSGALG00000021752)*

Differentially expressed or methylated genes in parents and offspring that are located <50Kb from selective sweeps.

# Novel transcript annotated with Affymetrix probset ID

** Novel gene annotated with Ensembl ID*
