# Supplementary material for: Heritable genome-wide variation of gene expression and promoter methylation between wild and domesticated chickens
Source: BMC Genomics. 2012 Feb 4;13:59. doi: 10.1186/1471-2164-13-59 (PMC3297523; doi:10.1186/1471-2164-13-59)
Supplement: Additional file 8 — Primer structures. The bisulfate converted primers used in HRM. [file 1471-2164-13-59-S8.PDF]

Additional file 8 | **Bisulfate converted primers used in HRM**

Genes	Forward	Reverse	Annealing (^{o}C)	Marker (Kb)*
FUCA1	TCGGTGTTAGGTTAGTGCGTAG	AACCCCATCCCCTCAAATAC	55	828
PCDHAC1	GCGTTAGCGAGGTGGGTAGTT	CACCATCAAATCGCTCTTACCC	55	176
RUFY3	TGAAAAGGGTAGATGTCGTATTGA	TCTCAAACTCCTTTATCAAACCAA	54	1449
TXNDC16	TTGTTAAAGTGTAGGACGGGGTTG	CCCCAACTACAAACAAAACACCA	54	1426

** Distance to genetic marker either for RJF or WL allele*
